# Neuron ID dataset facilitates neuronal annotation for whole-brain activity imaging of *C. elegans*

**DOI:** 10.1186/s12915-020-0745-2

**Published:** 2020-03-19

**Authors:** Yu Toyoshima, Stephen Wu, Manami Kanamori, Hirofumi Sato, Moon Sun Jang, Suzu Oe, Yuko Murakami, Takayuki Teramoto, Chanhyun Park, Yuishi Iwasaki, Takeshi Ishihara, Ryo Yoshida, Yuichi Iino

**Affiliations:** 1 Department of Biological Sciences, Graduate School of Science, The University of Tokyo, Bunkyo-ku, Tokyo, Japan; 2 The Institute of Statistical Mathematics, Research Organization of Information and Systems, Tachikawa, Tokyo Japan; 3 The Graduate University for Advanced Studies, SOKENDAI, Mishima, 411-8540 Japan; 4 Department of Biology, Faculty of Sciences, Kyushu University, Higashi-ku, Fukuoka, Japan; 5 Department of Mechanical Systems Engineering, Graduate School of Science and Engineering, Ibaraki University, Hitachi, Ibaraki Japan

**Keywords:** Neuron identification, *Caenorhabditis elegans*, Cell-specific promoters, Volumetric images, Large dataset for cell positions, Biological resources, Computational method, Whole-brain activity imaging

## Abstract

**Background:**

Annotation of cell identity is an essential process in neuroscience that allows comparison of cells, including that of neural activities across different animals. In *Caenorhabditis elegans*, although unique identities have been assigned to all neurons, the number of annotatable neurons in an intact animal has been limited due to the lack of quantitative information on the location and identity of neurons.

**Results:**

Here, we present a dataset that facilitates the annotation of neuronal identities, and demonstrate its application in a comprehensive analysis of whole-brain imaging. We systematically identified neurons in the head region of 311 adult worms using 35 cell-specific promoters and created a dataset of the expression patterns and the positions of the neurons. We found large positional variations that illustrated the difficulty of the annotation task. We investigated multiple combinations of cell-specific promoters driving distinct fluorescence and generated optimal strains for the annotation of most head neurons in an animal. We also developed an automatic annotation method with human interaction functionality that facilitates annotations needed for whole-brain imaging.

**Conclusion:**

Our neuron ID dataset and optimal fluorescent strains enable the annotation of most neurons in the head region of adult *C. elegans*, both in full-automated fashion and a semi-automated version that includes human interaction functionalities. Our method can potentially be applied to model species used in research other than *C. elegans*, where the number of available cell-type-specific promoters and their variety will be an important consideration.

## Introduction

Identification of the cell is an essential process in the broad fields of biology including neuroscience and developmental biology. For example, identification of cells where a gene is expressed can often be the first step in analyzing functions and interactions of the gene. Also, the identity information is required for comparing cellular activities across different animals. In order to annotate cell identities in microscopic images, features of the cells such as positions and morphologies are often compared between the samples and a reference atlas.

The nematode *Caenorhabditis elegans* has a unique property that all cells and their lineages have been identified in this animal [1, 2]. Additionally, the morphology and the connections between all 302 neurons in adult hermaphrodites were also identified by electron microscopy reconstruction [3]. Such detailed knowledge opens up unique opportunities in neuroscience at both single-cell and network levels. Recent advances in microscopy techniques also enable whole-brain activity imaging of the worm [4–11], even for the free-moving worms [12–14]. The neural activities were obtained at single-cell resolution, and the identities of limited numbers of neurons were annotated manually in some of the studies [4–8, 10, 14]. However, there is no systematic and comprehensive approach to annotate the neurons in whole-brain activity data [12].

Annotation of neuronal identities in *C. elegans* is often performed based on positions of the neurons, especially for larval animals in which the neurons are located at stereotyped positions [15, 16]. However, for adult animals, the positions of the neuronal cell bodies are highly variable between animals [12]. Several additional pieces of information can be used such as superimposed cell identity markers and morphological information of the neurons. Currently, superimposing cell identity markers, such as fluorescent proteins expressed by well-characterized cell-specific promoters, is the most popular and reliable method for neural identification. For example, Serrano-Saiz et al. showed that such methods are effective when the number of the target neurons is limited [17]. However, integrating this approach with the whole-brain activity imaging seems difficult because it requires different markers and different fluorescent channels for every neuron in principle. Morphological information is also useful when the number of target neurons is very limited, but it is not readily utilized for whole-brain imaging because the neurons are distributed densely in the head region of the worms and the morphological information cannot be obtained accurately.

Several efforts for developing automatic annotation methods were reported. In order to annotate the neurons based on their positions, the information of the positions and their variations will be required. Long et al. [18, 19] produced 3D digital atlas for 357 out of 558 cells from several tens of L1 animals, and related works also used the atlas [20, 21]. The atlas consists of positions and their deviations of the cell nuclei of body wall muscles, intestine, pharyngeal neurons, and neurons posterior to the retrovesicular ganglion, as well as some other cell types. However, the neurons anterior to the retrovesicular ganglion are omitted because of their dense distribution [19], and the atlas is not applicable to the neurons in the head region important for neural information processing. Aerni et al. [22] reported positions of 154 out of 959 cells from 25 adult hermaphrodites, including intestinal, muscle, and hypodermal cells, and introduced a method that integrates useful features including fluorescent landmarks and morphological information with the cell positions. Nevertheless, the positions of neurons were not reported. As far as we know, the information of the positions of the neurons in adult worms can be obtained only from the atlas produced by the EM reconstruction work [3]. Unfortunately, the White atlas does not have the information about the variety of the positions between individual animals. Additionally, the atlas may be deformed because of inherent characteristics of the sample preparation methods for electron microscopy. Thus, experimental data of positions of neurons in adult animals were very limited.

Here, we measure the positions of the neurons in adult animals by using cell-specific promoters and create a dataset. We evaluate the variations of the positions and obtain an optimal combination of the cell-specific promoters for annotation tasks based on accumulated information of cell positions. Our optimal strains enable rational manual annotation of most neurons in the head region of an animal. We also develop and validate an efficient annotation tool that includes both automated annotation and human interaction functionalities.

## Results

### A neuron ID dataset of head neurons

**Fig. 1:**
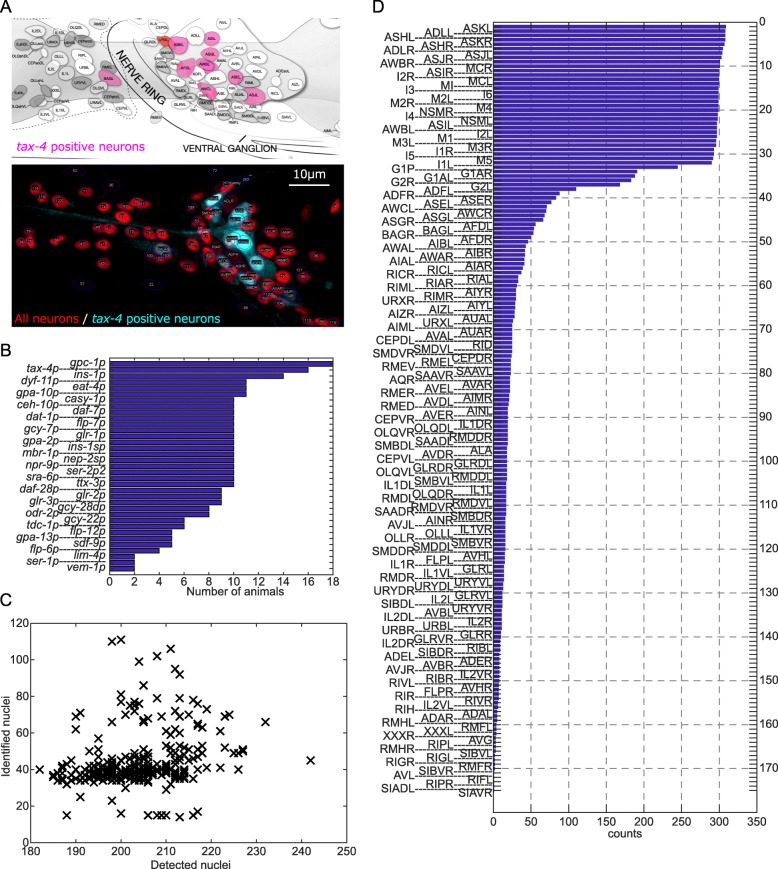
Outline of the neuron ID dataset. **a** The expression pattern of cell-specific promoter *tax-4p* (modified from WormAtlas) and an example image of the strain JN3006 in which the landmark fluorescent protein was expressed by *tax-4p*. The maximum intensity projection of the right side of a representative animal is shown. **b** The list of the cell-specific promoters and the number of animals used in the neuron ID dataset. **c** The number of the detected and the identified nuclei in each animal. **d** The names of identified cells and the number of animals (“counts”) in which the cells were identified. The names and the positions of all identified cells in each animal were summarized in Figshare Dataset S1 [23] and Additional file 1: Table S1

In this study, we focused on the head neurons of an adult animal of the soil nematode *C. elegans*, which constitute the major neuronal ensemble of this animal [3]. The expression patterns of cell-specific promoters were used as landmarks for cell identification (Fig. 1a). The fluorescent calcium indicator Yellow-Cameleon 2.60 was expressed in a cell-specific manner by using one of the cell-specific promoters and used as a fluorescent landmark. All the neuronal nuclei in these strains were visualized by the red fluorescent protein mCherry. Additionally, the animals were stained by a fluorescent dye, DiR, to label specified 12 sensory neurons following a standard method [24]. The worms were anesthetized by sodium azide and mounted on the agar pad. The volumetric images of the head region of the worm were obtained with a laser scanning confocal microscope. All the nuclei in the images were detected by our image analysis pipeline roiedit3D [10] and corrected manually. The nuclei were annotated based on the expression patterns of fluorescent landmarks.

Finally, we obtained volumetric images of 311 animals with 35 cell-specific promoters in total (Fig. 1b). On average, 203.7 ± 0.52 (mean ± standard error) nuclei were found and 44.2 ± 0.86 (mean ± standard error) nuclei were identified (Fig. 1c). The names and the positions of all identified cells in each animal were summarized in Figshare Dataset S1 [23]. The names of identified cells in each strain are also summarized in Additional file 1: Table S1. These positions and promoter expression information are hereafter called the neuron ID dataset.

Figure 1d shows the names of identified cells and the number of animals (“counts”) in which the cells were identified. In most animals, 12 dye-stained cells and 25 pharyngeal cells were identified, in addition to cells identified by using cell-specific promoters. Finally, we identified a total of 175 out of 196 cells ranging from I1 class neurons (anterior) to AVG neuron (posterior). Out of 182 cells anterior to the retrovesicular ganglion, 171 cells were identified. We did not identify 11 cells including URA class (4 cells), RIS cell, SIA class (2 of 4 cells), AVK class (2 cells), and RMG class (2 cells) because of the lack of suitable cell-specific promoters. We also identified 4 cells in the retrovesicular ganglion including AVG cell, RIF class (1 of 2 cells), and RIG class (2 cells). This result indicates the neuron ID dataset covers a majority of neurons in the head region.

Note that we used H20 promoter as a pan-neuronal promoter [25]. We confirmed H20 promoter was expressed in the GLR glial cells and XXX atypical hypodermal cells by co-expressing the cell-specific promoters *nep-2sp* and *sdf-9p*, respectively. We estimated that H20 promoter was also expressed in pharyngeal gland cells and HMC cell, based on their positions. Also, we estimated that H20 promoter was expressed weakly in the hypodermal cells, based on their positions and shape of the nuclei, but we removed HMC and hypodermal cells from our neuron ID dataset. We confirmed H20 promoter was not expressed in the socket cells nor the sheath cells by co-expressing the cell-specific promoter *ptr-10p*.

### Large variation disrupts position-based cell annotation

How large is the variation of the relative positions of the cells between individual animals? To answer this question, we need to first assess the potential sources of the variation. Intuitively, there are several possibilities: (1) placement (translational and rotational) of the worms in the obtained images, (2) curved posture of the worms (body bending), and (3) inherent variation of the cell position. In order to focus on the inherent variation that we are interested in, we considered a few ways to remove the contribution of (1) and (2). Principal component analysis (PCA) and subsequent alignment processes corrected the translation and rotation (see the “Methods” section). The quadratic curve fitting was employed to correct curved posture (Additional file 2: Figure S1). These methods significantly reduced the variation of cell positions (Additional file 3: Figure S2, Additional file 4: Table S2). Note that, after posture correction, the cell distribution is not deformed in the DV-LR plane (Additional file 2: Figure S1C, right panel), indicating that the distortion of worms is negligibly small under our experimental setups. To assess the contribution of temporal variation of cell positions in each animal, we utilized the time-lapse images obtained for whole-brain imaging. A large part of the movement of cells during time-lapse imaging can be removed by translation correction, and the remaining movements are too small to explain the observed variation of cell positions in the neuron ID dataset (Additional file 5: Figure S3, Additional file 6: Table S3).

**Fig. 2:**
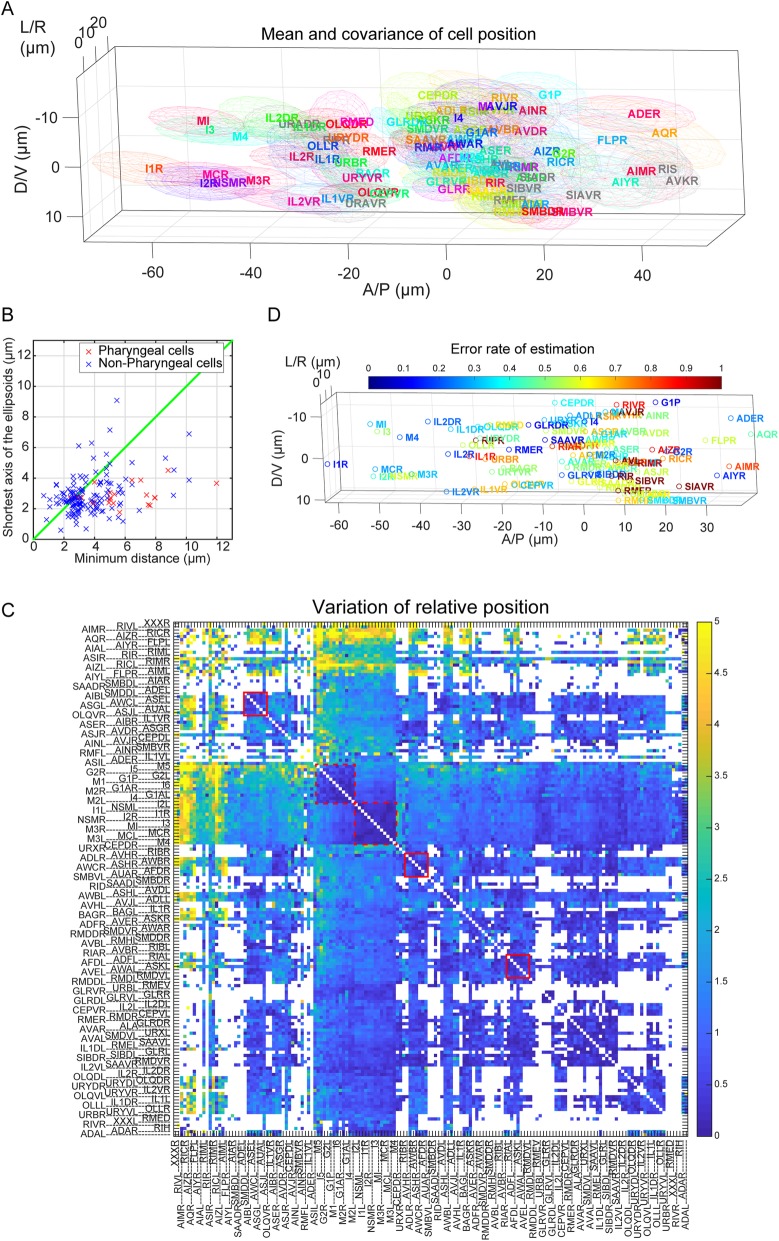
Variations of cell positions. **a** Visualization of the variation of cell positions. The ellipsoid indicates the mean and the covariance of the positions of the cells. Cells in the right half of the body are shown. The colors are assigned randomly for visualization. In the case of the cells whose covariance cannot be calculated, the median of other covariance was used for visualization and shown in gray color. A/P means anterior-posterior, D/V means dorsal-ventral, and L/R means left-right directions. **b** Minimum distance (Euclid distance of centers of nearest ellipsoids) and the shortest axis length of the ellipsoids (equal to the twice of the smallest standard deviation) for each cell. The line shows where the minimum distance equals the shortest axis length. **c** Variation of the relative position of cell pairs is shown as a heat map. The red box and red dotted box indicate clusters of less varying cell pairs in the lateral ganglion and pharynx, respectively. For visualization, the variations were divided by their median value, and the color axis was truncated at 5 (the colors for cell pairs whose variation is larger than 5 are the same as the color for cell pairs whose variation is 5). **d** The error rate of the naive estimation method is visualized with cell positions in 3D. In the naive estimation method, the posterior probability of assignments was calculated for the respective cells in the respective animals based on the mixture of the Gaussian distributions. The name of the cell was estimated as the name of the Gaussian which had the largest probability for the cell. The error rates were calculated for each ground-truth cell. The hot color indicates that the error rate is high

After removing the contribution of (1) and (2), we compiled the positions of the named cells in the neuron ID dataset. The positions of the nuclei identified as the same cell were collected from the neuron ID dataset (Additional file 7: Figure S4). The mean and the covariance of the positions specify a tri-variate Gaussian distribution, which can be considered as the maximum likelihood estimate of cell position distribution. The three-dimensional ellipsoidal region of 2 standard deviation of the tri-variate Gaussian distribution is shown for each cell, in which about 70% of data points are expected to be included (Fig. 2a). The ellipsoids largely overlap with each other, especially in the lateral ganglia (mid region of the head), because of high variation and high density of the cells. The median distance between distribution centers of neighboring cells was 3.19 ± 0.15 μm (median ± standard error) (Fig. 2b). The median length of the shortest axis of the ellipsoids, equivalent to the twice of the smallest standard deviation, was about 2.69 ± 0.09 μm (median ± standard error). The values of the minimum distance and the shortest axis length were almost the same, indicating that the variation of the position of a cell reaches the mean position of the neighboring cells. Thus, the variations of the cell positions between individual animals are large.

The variations of the cell positions were explored further in a different way. We focused on the variations of the relative positions of neuron pairs. If we fix the position of a cell and align all other cells, the specific-cell-centered landscape can be drawn (Additional file 8: Figure S5). When ASKR cell was centered, the variations of positions of adjacent cells including SMDVR and ADLR were decreased, but that of other cells did not change or rather increased. When MI cell, an anterior pharyngeal neuron, was centered, the variations of positions of pharyngeal cells decreased, but those of other cells generally increased. These results suggest that the variations of relative positions are different depending on neuron pairs. We further obtained the variations of relative positions of all available cell pairs (Fig. 2c). The volume of the ellipsoid of relative positions is regarded as the variation of relative positions. We found clusters of less varying cell pairs (Fig. 2c, red boxes). The clusters include lateral ganglion cell pairs and pharyngeal cell pairs (Additional file 9: Figure S6). On the other hand, there are highly varying cells including RIC, AIZ, and FLP classes.

Where do these variations of cell positions come from? In order to tackle this problem, we performed an additional analysis. The pharynx of worms moves during development, and the position of the pharynx in the anterior-posterior axis differs between individual animals. We found that the positions of dye-positive cells were affected by the positions of the pharynx (Additional file 10: Figure S7, Additional file 11: Table S4); when the pharynx moved anteriorly, all the dye-positive cells (ASK, ADL, ASI, AWB, ASH, and ASJ classes) moved outside in lateral positions. In addition, anterior cells (ASK, ADL, and AWB classes) moved anteriorly, and posterior cells (ASJ class) moved posteriorly. In other words, the pharynx of worms pushed these neurons aside. This result indicates that some part of the inherent variations of cell positions come from the variations in organ placement.

How does the variations of cell positions disrupt the position-based cell annotation? Based on the mixture of the Gaussian distributions (Fig. 2a), the posterior probability of assignments was calculated for the respective cells in the respective animals. The name of the cell was estimated as the name of the Gaussian distribution which has the largest probability at the position of the cell in the target animal. In other words, the cell has the probabilities in which the cell belongs to the Gaussian distributions, and the most likely distribution was assigned for the cell. The error rates of this estimation method were visualized with cell positions (Fig. 2d). The error rates for the cells anterior to the nerve ring were relatively low (44.5% ± 24.0%, mean ± standard deviation), and those for the cells in the ventral ganglion were relatively high (64.8% ± 25.6%, mean ± standard deviation). Mean error rate was 49.7% ± 24.3% (mean ± standard deviation) (see Fig. 4c, described below), indicating that the variations of the cell positions actually disrupt the position-based cell annotation severely.

### Optimal combination of the cell-specific promoters increases the number of identified cells in an animal

In order to reduce the error rate of the annotation method, one may want to use the information of fluorescent landmarks [8, 12]. Using multiple landmarks will reduce the error rate. One or two fluorescent channels are often available for the landmarks in addition to the channels required for the whole-brain activity imaging. We therefore sought for the optimal combination of cell-specific promoters for two-channel landmark observation using the neuron ID dataset.

**Fig. 3:**
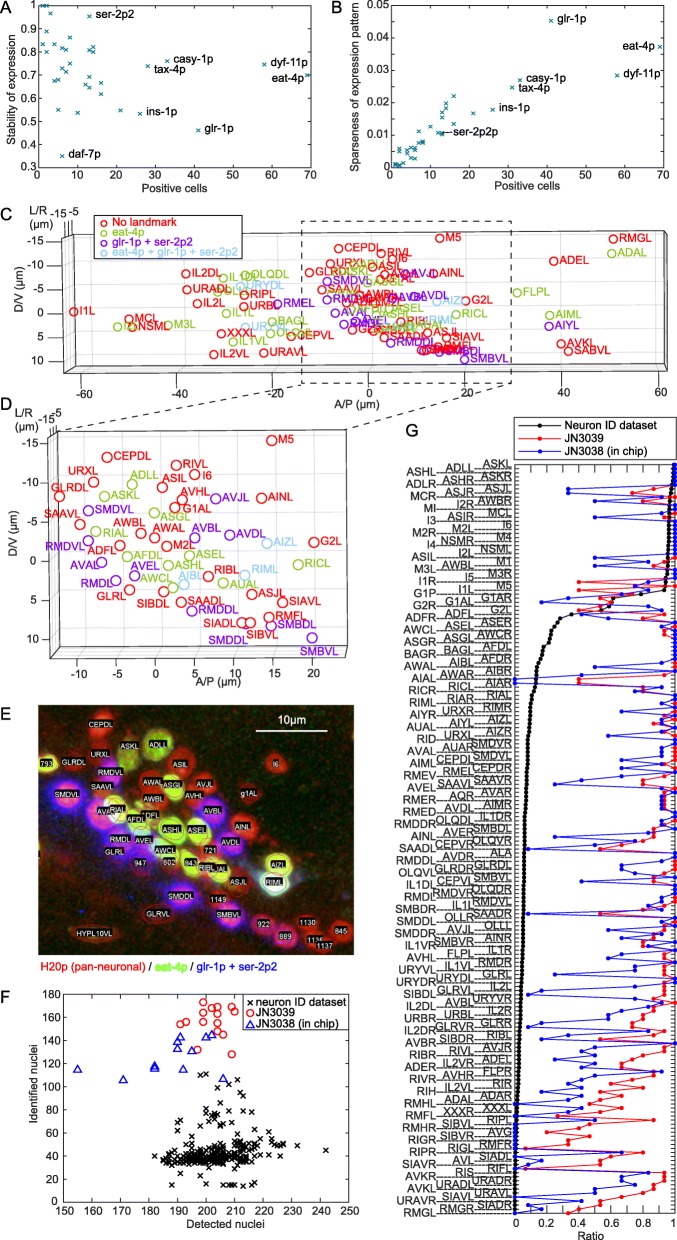
Optimal combination of the cell-specific promoters increases the number of identified cells in an animal. **a** Number of positive cells and stability of expression of cell-specific promoters. A cell was counted as positive for a promoter if the cell expresses the landmark fluorescent protein in at least one animal. The positive ratio is a ratio of positive (expressing) cells over the total number of the cells (= number of tested animals). Stability of expression was calculated as an average of the positive ratio over the cells in which at least one cell is positive for the promoter. **b** Number of positive cells and sparseness of the expression pattern (see the “Methods” section) of the cell-specific promoters. Note that, for visibility, only the several labels are shown in **a** and **b**. Fully labeled panels are shown in Additional file 12: Figure S8. **c** Visualization of the optimal combination of the cell-specific promoters. The cells in the right half of the body are shown. **d** A part of **c** is zoomed for comparison with **e**. **e** An example fluorescent image of JN3039 strain and annotated cell names. See Additional file 14: Figure S9 for enlarged image. **f** The number of the detected and the identified nuclei in each animal. **g** The names of identified cells and the identification ratio, which is a ratio of identified cells over the total number of the cells. The solid lines are guide for visualization

Several properties of the promoters were evaluated in order to choose the optimal combination: the number of cells that are labelled (Fig. 3a, b, Additional file 12: Figure S8), stability of expression (Fig. 3a), sparseness of the expression pattern (Fig. 3b, see the “Methods” section for definition), and overlap of expression patterns in the case of combinations (see Figshare Dataset S1 [23]). Among the 35 tested promoters, *eat-4p* was selected because it was expressed in the most numerous cells in the head region (Fig. 3a). The promoters *dyf-11p* and *glr-1p* were also expressed in numerous cells, and *glr-1p* was selected as the second promoter because the sparseness of the expression patterns of *glr-1p* was higher than that of *dyf-11p* (Fig. 3b) and because the expression patterns of *dyf-11p* highly overlapped with that of *eat-4p*. Additionally, *ser-2p2* was selected based on the stability of the expression and low overlaps with *eat-4p* and *glr-1p*. Thus, the combination of *eat-4p*, *glr-1p*, and *ser-2p2* was selected (Fig. 3c). The latter two promoters were used with the same fluorescent protein assuming only two fluorescent channels can be used for the landmarks as is the case for our experimental setup for whole-brain imaging. In the neuron ID dataset, *eat-4p* was expressed in 69 cells and *glr-1p + ser-2p2* were expressed in 50 cells out of 196 cells in the head region of adult worms.

All combinations of the promoters could be evaluated by an algorithm that considers the number of expression, sparseness, and overlap of expression patterns (see the “Methods” section and Additional file 13: Table S5). In brief, the algorithm highly evaluated a combination when two neighboring cells were in different colors. In the case of three promoters and two fluorescent channels, the combination consisting of *eat-4p*, *glr-1p*, and *ser-2p2* was placed in the 18th rank out of the possible 20,825 combinations.

Here, we produced a strain JN3039 as follows. The far-red fluorescent protein tagRFP675 was expressed using *eat-4p*, and the blue fluorescent protein tagBFP was expressed using *glr-1p* and *ser-2p2*. The red fluorescent protein mCherry was expressed using the pan-neuronal promoter H20p. This strain does not use fluorescent channels of CFP, GFP, and YFP and is useful for the cell identification tasks. For example, if there is a strain that expresses one of these fluorescent proteins with a promoter whose expression patterns need to be identified, one can do it by just crossing the strain with our standard strain JN3039.

With the help of the optimized expression of landmark fluorescent proteins in JN3039, the number of identified cells in an animal is expected to increase compared to the strains used to make the neuron ID dataset. Actually, by using the JN3039 strain, we could manually identify 162.5 ± 4.23 (mean ± standard error) nuclei out of 202 ± 1.46 (mean ± standard error) detected nuclei from 15 adult animals (Fig. 3e–g, Additional file 14: Figure S9, Additional file 15: Dataset S2). Ignoring hypodermal cells reduced the number of the identified nuclei to 156.3 ± 3.46 (mean ± standard error). The number of identified cells in the latter case is 3.6 times higher than the average of the neuron ID dataset. We identified a total of 186 out of 196 cells that covers most neurons in the head region (Fig. 3g). These results indicate that our optimized strain is a powerful tool for neuronal annotation, for example for identifying expression patterns of genes of interest.

Additionally, a strain JN3038 was made from the strain JN3039 by expressing fluorescent calcium indicator Yellow-Cameleon 2.60 with the pan-neuronal promoter H20p. This five-colored strain inherits the neuron identification ability of JN3039 and will enable whole-brain activity imaging with annotation. We could manually identify 130.3 ± 4.63 (mean ± standard error) nuclei out of 188 ± 4.08 (mean ± standard error) detected nuclei on average from 12 adult animals (Fig. 3f, g). In total, we identified 171 out of 196 cells. The numbers of identified and detected nuclei were slightly lower than JN3039, likely because of the difference of experimental conditions including resolution of the optics and photobleaching.

We tested the health of JN3038 animals because worms often get sick by introducing multiple transgenes. The JN3038 animals displayed reduced brood size and moved slowly (Additional file 16: Figure S10A-D, Additional file 17: Table S6). Further, we tested whether the animals show normal salt chemotaxis. On a plate with NaCl gradient, worms are attracted to the NaCl concentrations which they have experienced with food [26]. Additionally, they avoid the NaCl concentrations which they have experienced without food [27]. Thus, the salt chemotaxis behavior is a kind of associative learning. Studying the neural mechanism of the behavior will also elucidate that of the learning. The JN3038 animals showed salt chemotaxis and learning ability similar to wildtype animals (Additional file 16: Figure S10E). This result suggests the neural circuit of the animals is healthy enough so that we will be able to dissect neural mechanisms of the learning and the behaviors by using the JN3038 strain.

### Annotation algorithm in the computer-assisted semi-automatic annotation framework

Although most neurons in the head region of an animal could be annotated successfully by using our optimized strain, the manual annotation task often takes a long time and requires high expertise. Automatic methods for neural identity annotation will reduce the difficulty. However, such methods will suffer from low accuracy because the positions of the cells show large variations that disrupt the performance of a position-based cell annotation (Fig. 2d). Therefore, we developed an automatic annotation method as a part of a computer-assisted semi-automatic annotation framework. Note that our aim is not to establish a fully automatic annotation method, which will be extremely difficult, but to provide a computer-assisted semi-automatic annotation framework in which the accuracy can be improved through human-machine interaction. An example usage is an ab initio automatic estimation followed by manual correction. The computer estimation can also be used as a help for a decision during manual annotation.

**Fig. 4:**
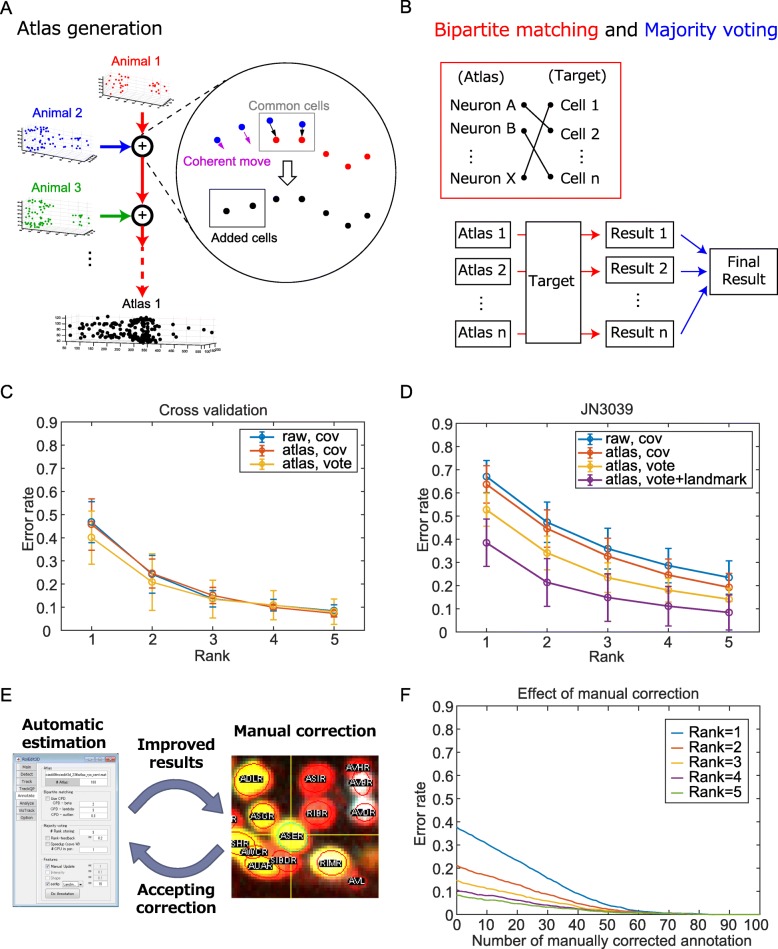
An automatic annotation method and evaluation. **a** The outline of the atlas generation method. **b** The outline of the automatic annotation method. The schemes of bipartite graph matching and majority voting are shown. **c** Error rates of the automatic annotation method for the animals in the neuron ID dataset. The names of the cells were estimated based on their positions. The error rate was calculated as 1 – (*N*_correct_)/(*N*_annotated_) for each animal, where *N*_annotated_ is the number of human-annotated cells (ground truth) and *N*_correct_ is the number of cells whose annotation by the algorithm was correct. Cells un-annotated by human were not included in the calculation of error rate. The rank *R* indicates that it is considered correct if the correct annotation appeared in the top *R* estimations by the algorithm. The error rates were evaluated by cross-validation, and mean ± standard deviation over well-annotated six animals is shown. **d** Error rates of the automatic annotation method for the strain JN3039 that expresses the fluorescent landmarks. The names of the cells were estimated based on their positions with or without the expression of landmark promoters. Mean ± standard deviation over 15 animals is shown. **e** The automatic annotation method was integrated in the graphical user interface roiedit3d that enables feedback between automatic and manual annotations. **f** The effect of manual correction on the error rate of automatic annotation. A wrong annotation of a cell in rank 1 estimation for JN3039 (see Fig. 4d) was corrected, and the automatic annotation method was performed by using the correction information. This step was repeated sequentially

The proposed annotation method consists of three parts: the generation of a large set of atlases (Fig. 4a), bipartite matching, and majority voting (Fig. 4b). The generated atlases capture high-order information of positional variations in the neuron ID dataset. During the bipartite matching step, the positional information is integrated with the additional information including expressions of landmark promoters and human correction. Majority voting reduces the effect of positional variations by exploiting the large set of generated atlases and returns multiple candidates that will reduce the difficulty of the manual annotation.

In order to generate an atlas with fully annotated cells, we combine positional information of cells from multiple partially annotated animals while maintaining the relative position between the cells as much as possible. We assembled the partially identified cell positions in our neuron ID dataset as follows (Fig. 4a, see the “Methods” section for detail):

A) Choose an animal 1 and extract the positions of the identified cells.
B) Choose an animal 2 and register it to animal 1 based on the positions of commonly identified cells in both animals.
C) Map the cells in animal 2 that were not identified in the animal 1 according to the registration, and add them to the identified cell list of animal 1.
D) Repeat steps B and C until all the animals were covered.
E) Add the positions of cells that were not identified in our neuron ID dataset using the positions of the cells in White’s atlas.

The resulting atlas depends on the order of assembly, which reflects the variation of the cell positions between individual animals. By random sampling of the animals, we generated around 3000 synthetic atlases that were used to reduce the error rate of the estimation.

In the bipartite matching step, the cells in a target animal were assigned to those in an atlas. An assignment of a cell in the target animal to the one in the atlas has a cost based on the similarity (or dissimilarity) of the two cells. The similarity scores are based on several factors including but not limited to Euclidean distance, expressions of landmark promoters, and the feedback from human correction as needed. The optimal combination of the assignments that minimize the sum of the costs was obtained by using the Hungarian algorithm. The name of the cell in the target animal can be estimated as the name of the assigned cell in the atlas in this step.

In the majority voting step, the bipartite matching of the target animal was repeated with different atlases. Each assignment of a cell is considered as a vote, and the most voted assignment was considered as the top rank estimation of annotation. Assuming the generated atlases could capture the positional variations of the cells, the vote of erroneous estimations will be dispersed and the vote of correct estimation will be a top rank.

In order to validate our atlas generation, we compared the position of cells in the generated atlases and the dataset. We visualized the mean and the covariance of the positions of the cells in the atlases (Additional file 18: Figure S11A). The outline of the cell positions in the atlas was similar to that in the dataset, suggesting that our atlases capture the positional variations of the cells in the dataset. The distances of mean positions of the cells between the atlases and the dataset were small (Additional file 18: Figure S11B, 1.43 μm in median), but became large for the rarely detected cells. The covariance of the positions of the cell between the atlas and the dataset was also similar (Additional file 18: Figure S11C), but the covariance for the atlases was larger than that for the dataset when the cell was rarely detected in the dataset. The variation of relative positions of the cell pairs in the atlases was similar to that in the dataset when the cell pair was co-detected in the dataset frequently enough, and became larger when the cell pair was less co-detected (Additional file 18: Figure S11D and Additional file 19: Figure S12). These results indicate that our atlases capture the positional information of the cells and its variations with high-order information (i.e., the variation of relative positions of the cell pairs). These results also suggest the atlases will be more precise when the neuron ID dataset includes a lot more animals and annotated cells.

To validate our automatic annotation method, a five-fold cross-validation test was performed. All the animals in the neuron ID dataset were randomly divided into five subsets. We performed a total of five tests. For each test, we excluded one of the subsets and used the remaining subsets for generation of atlases and used it to estimate the annotation performance of the reserved set based on the generated atlases. The error rate of bipartite matching was relatively high, and the majority voting could deliver significant improvements of the annotation accuracy (Additional file 20: Figure S13). On average, 78.8% ± 6.71% (mean ± standard error) nuclei were annotated and 46.2 ± 2.73 (mean ± standard error) nuclei were successfully estimated as the top rank, and the error rate of the top rank estimation was 40.1% ± 4.69% (mean ± standard error) (Fig. 4b, c). As a control, two methods were introduced; one method only considered the mean and covariance of the cell positions of raw data (without using the atlases and voting, see Fig. 2d). The other method considered the mean and covariance of the cell positions in the atlases (without using majority voting). The error rates of the two methods were higher than those of the proposed method, indicating that the majority voting step in the proposed method contributes to the correct estimation (Fig. 4c). If we considered the accuracy for the top 5 voted estimations (shown as rank 5), the error rate decreased to 8.09% ± 2.24% (mean ± standard error).

The automatic annotation method was applied to the animals with fluorescent landmarks (strain JN3039, see Fig. 3c–e). The error rates of the top rank estimation with and without fluorescent landmarks were 38.5% ± 2.64% (mean ± standard error) and 52.7% ± 1.84% (mean ± standard error), respectively, indicating that utilizing the fluorescent landmarks also contributes to the correct estimation (Fig. 4d). If we considered the accuracy for the top 5 voted estimations, the error rate decreased to 8.40% ± 1.95% (mean ± standard error). These error rates were comparable to the cross-validation results for the neuron ID dataset, suggesting that our annotation framework will work correctly for the whole-brain activity imaging.

In our neuron ID dataset, dye-positive 12 neurons and 25 pharyngeal cells were overrepresented because of our neuron identification strategy. We confirmed that this imbalance had little impact on the error rate of the automatic annotation for cells identified in JN3039 (Additional file 21: Figure S14), indicating the robustness of our automatic annotation method.

The automatic annotation method was also applied to the animals in a microfluidic chip for whole-brain activity imaging (Additional file 22: Figure S15). The error rates of the top rank estimation with and without fluorescent landmark were 52.1% ± 2.10% (mean ± standard error) and 72.8% ± 2.16% (mean ± standard error), respectively, and that of the top 5 voted estimations was 12.2% ± 1.32% (mean ± standard error). The worms were compressed and distorted to be held in the microfluidic chips, and the distortion of the worm may increase the error rates. During whole-brain imaging of free-moving animals [12–14]), the worms will be less compressed and less distorted, and our algorithm may work better.

Additionally, our algorithm was implemented in the GUI roiedit3d [10], and it can handle feedback information from the human annotations (Fig. 4e). One can choose the correct annotation from several estimated candidates. Once annotations are corrected manually, our automatic annotation method can accept corrections and can use them to improve the annotation of other neurons as well, because assignments inconsistent with manual annotation are not allowed in the bipartite graph matching step in our algorithm. Thus, our semi-automatic framework reduces the difficulty of annotation tasks. For another example, one can identify neurons manually by using other information including the neural activity or morphology; then, the automatic estimation for the other neurons will be improved. The final results can be added to the neuron ID dataset, and the annotation algorithm will work more accurately. Thus, the feedback system incorporates tacit knowledge into the automatic annotation method. A detailed step-by-step tutorial for semi-automatic annotation using RoiEdit3D is provided as Additional file 23: Dataset S3.

Finally, we tested the effect of the manual correction to the error rate of the automatic annotation method. We randomly selected a wrong annotation of a cell in rank 1 estimation for JN3039 (see Fig. 4d), corrected the annotation, and re-ran the automatic annotation method using the manual correction information. This step was repeated sequentially. The error rate decreased to zero when the number of the manual correction increased (Fig. 4f). Manual correction of one cell label led to 1.078 ± 0.0369 (mean ± standard error) increase in the correct cell labelling on average. This result indicates that our computer-assisted semi-automatic annotation framework corrected an additional 7.8% estimation by using manual correction information. Similarly, we tested the effect of prior manual annotation on the correct rate of automatic annotation. Annotation of a single cell type was specified manually before performing the automatic annotation for cells identified in JN3039 (Additional file 24: Figure S16 and Additional file 25: Figure S17). Prior manual annotation of SMDVL gave the largest improvement of the correct rate (0.59%, about 2 cells). Thus, through the interactive process, our algorithm will make human annotation tasks more efficient.

## Discussion

In this study, we obtained volumetric fluorescent images of 311 animals using 35 promoters and created a neuron ID dataset that contained the positions of the identified cells and expression patterns of promoters in the respective animals. Utilizing the neuron ID dataset, we evaluated the variation of the positions of the cells and demonstrated the difficulty of performing accurate annotation of the neurons. Based on statistical analyses using our neuron ID dataset, we chose the combination of the promoters optimal for our annotation tasks. The optimal strains we produced enabled rational annotation of most head neurons in an adult animal. We proposed a semi-automatic annotation method and validated its performance on head neurons of adult worms for whole-brain imaging. Thus, we successfully integrated the annotation techniques with the whole-brain activity imaging.

The cell positions of real animals and their variation are the most important information for cell identification. As far as we know, this study is the first report about the large-scale information of the positions of the cells in the head region of adult *C. elegans*, which lead to the systematic and comprehensive method to the annotation of the head neurons. On the other hand, the neuron ID dataset is still incomplete. Several cells were underrepresented or not detected in our dataset (Fig. 1d). We found the number of detection counts was one of the factors contributing to the error rate of the automatic annotation method (Additional file 21: Figure S14). The additional measurement for the neuron ID dataset will improve the error rate of the automatic annotation method.

Through the analysis of the neuron ID dataset, we found that the variation of cell positions is very large. Although we carefully removed the deformation caused by the posture of worms by using quadratic transformation, we cannot show our alignment methods were able to remove such deformation completely. While elastic transformation methods such as thin plate spline [28] and coherent point drift [29] could be used for registering cell positions, the effect will probably be limited because the variability in cell positions are non-uniform. We found inter-animal variability was larger than temporal differences (i.e., intra-animal variability) in our experimental setups. This result suggested that the inter-animal variability rather than intra-animal variability was the main cause of the errors in the automatic annotation.

The deformation of worms in the microfluidic chip, which is used for observing the response of neurons to sensory stimuli, might increase the variation of cell positions and affect the error rate of the automatic annotation. Measuring and accumulating the position of cells in animals under the microfluidic chip may decrease the error rate of automatic annotation if the deformation by the chip is common between animals.

The design of automatic annotation methods is based on the analysis of the neuron ID dataset. We found some co-varying neuron pairs (Additional file 9: Figure S6), but higher-order (i.e., triplet or more) co-variations were not captured explicitly in this paper. The atlas synthesis process in our automatic annotation method was designed to include these co-varying neuron pairs and higher-order information in the neuron ID dataset even if the information was not captured explicitly. We developed a comprehensive method to implicitly handle such variation through atlas generation and annotation based on majority voting. On the other hand, the automatic annotation method introduced in the ongoing neuroPAL project [30] utilized fluorescence intensity information, which can be a positive addition to our method to further improve the overall annotation performance.

Cell identification mainly relies on cell position and fluorescence information. Considering the fluorescence information, our optimal strain increased the number of identified cells in an animal to 156.3 cells on average. This covers 79.7% of 196 neurons and neuron-like cells (including XXX atypical hypodermal cells, GLR glial cells, and pharyngeal gland cells) located in the head region. Although this was a dramatic improvement compared to single promoters in the neuron ID dataset, a 100% cover rate was not achieved in this study. In order to improve the cover ratio, increasing the number of fluorescent channels and landmarks will be important. Long-Stokes shift fluorescent proteins might be good candidates because they use irregular fluorescent channels that will not be used in standard application. Actually, CyOFP1, one of the long-Stokes fluorescent proteins [31], was used in the neuroPAL project [30]. In our case, however, these proteins disrupted the neighboring fluorescent channels by leaking out. Employing color deconvolution techniques will increase the number of substantial fluorescent channels and may improve the cover rate, as well as the accuracy of the annotation algorithm.

The images of the animals we recorded will have useful information for annotation including the size of the nuclei and intensities of the fluorescence. In the manual annotation process, we utilized these pieces of information for improving the accuracy. On the other hand, our automatic annotation algorithm did not utilize these pieces of information and it may be one of the causes of relatively low accuracy of the algorithm. Recent advances in artificial neural networks especially in the field of image analysis will enable utilization of such information for automatic annotation. It is well known that artificial neural networks require a large amount of training data composed of images and the corresponding ground truth. Our neuron ID dataset contains images with identity information and will be ideal for the training data, but the number of data may not be enough. Our method that makes annotation more efficient will play an important role for opening up the path to the utilization of artificial neural networks in the future.

There is no dataset of cell positions that can be used as a benchmark of cell identification methods. For example, a new method that solves the cell identification problem as a nonlinear assignment problem was reported recently [32]. The proposed method was performed only on synthetic data. To evaluate the real performance of annotation methods, the methods should be tested on real data. Our neuron ID dataset will be an ideal benchmark of newly developed cell identification methods. Thus, our study will facilitate the future studies for automatic annotation methods.

The gene expression patterns often vary between individual animals. Although the degree of variation is important information especially for the neuron identification problems like this study, there are no quantitative data for variation of promoter expression patterns. Our dataset includes the probability of promoter expression in a cell with multi-channel volumetric images of individual animals. Additionally, recent works utilizing single-cell RNA sequencing techniques also revealed the variation of expression patterns of “cell-specific” genes [33, 34]. These data will form quantitative knowledge of variation of promoter expression patterns.

In order to identify the expression patterns of promoters, the most accurate method is testing whether the fluorescence of the promoter-driven fluorescent protein overlaps with the fluorescence of neuronal identity markers [17]. In such cases, our standard strain and automatic annotation method will help the selection of markers through objective estimation of cell identities.

Our framework of creating the neuron ID dataset and developing automatic annotation method can be applied to species other than *C. elegans*. For covering all neurons, the number of available cell-type-specific promoters and their variety will be important.

## Methods

### Strains and cultures

*C. elegans* strains used in this study are listed in Table 1. The sequences of promoters are summarized in Additional file 26: Dataset S4. Animals were raised on nematode growth medium at 20 °C. The *E. coli* strain OP50 was used as a food source.

**Table 1:** Strain list used in this study

Strain	Genotype	Used in
Bristol N2	*C. elegans* wild isolate	Figure S10
JN3000	*Ex[casy-1p::nls::YC2.60, lin-44p::GFP]; peIs2100[H20p::nls4::mCherry]*.	Figs. 1, 2, 3, and 4
JN3001	*Ex[ceh-10p::nls::YC2.60, lin-44p::GFP]; peIs2100[H20p::nls4::mCherry]*.	Figs. 1, 2, 3, and 4
JN3002	*Ex[daf-28p::nls::YC2.60, lin-44p::GFP]; peIs2100[H20p::nls4::mCherry]*.	Figs. 1, 2, 3, and 4
JN3003	*Ex[daf-7p::nls::YC2.60, lin-44p::GFP]; peIs2100[H20p::nls4::mCherry]*.	Figs. 1, 2, 3, and 4
JN3004	*Ex[dat-1p::nls::YC2.60, lin-44p::GFP]; peIs2100[H20p::nls4::mCherry]*.	Figs. 1, 2, 3, and 4
JN3005	*Ex[dyf-11p::nls4::YFP, lin-44p::GFP]; peIs2100[H20p::nls4::mCherry]*.	Figs. 1, 2, 3, and 4
JN3006	*Ex[eat-4p::nls::YC2.60, lin-44p::GFP]; peIs2100[H20p::nls4::mCherry]*.	Figs. 1, 2, 3, and 4
JN3007	*Ex[eat-4p::svnls2::TagRFPsyn;lin-44p::GFP]; peIs2100[H20p::nls4::mCherry]*.	Figs. 1, 2, 3, and 4
JN3008	*Ex[flp-6p::nls::YC2.60, lin-44p::GFP]; peIs2100[H20p::nls4::mCherry]*.	Figs. 1, 2, 3, and 4
JN3009	*Ex[flp-7p::nls::YC2.60, lin-44p::GFP]; peIs2100[H20p::nls4::mCherry]*.	Figs. 1, 2, 3, and 4
JN3010	*Ex[flp-12p::nls::Venus, lin-44p::mCherry]; peIs2100[H20p::nls4::mCherry]*.	Figs. 1, 2, 3, and 4
JN3011	*Ex[gcy-22p::nls::YC2.60, lin-44p::GFP]; peIs2100[H20p::nls4::mCherry]*.	Figs. 1, 2, 3, and 4
JN3012	*Ex[gcy-28p::nls::YC2.60, lin-44p::GFP]; peIs2100[H20p::nls4::mCherry]*.	Figs. 1, 2, 3, and 4
JN3013	*Ex[gcy-7p::nls::YC2.60, lin-44p::GFP]; peIs2100[H20p::nls4::mCherry]*.	Figs. 1, 2, 3, and 4
JN3014	*Ex[glr-1p::nls::YC2.60, lin-44p::GFP]; peIs2100[H20p::nls4::mCherry]*.	Figs. 1, 2, 3, and 4
JN3015	*Ex[glr-2p::nls::YC2.60, lin-44p::GFP]; peIs2100[H20p::nls4::mCherry]*.	Figs. 1, 2, 3, and 4
JN3016	*Ex[glr-3p::nls::YC2.60, lin-44p::GFP]; peIs2100[H20p::nls4::mCherry]*.	Figs. 1, 2, 3, and 4
JN3017	*Ex[gpa-2p::nls::YC2.60, lin-44p::GFP]; peIs2100[H20p::nls4::mCherry]*.	Figs. 1, 2, 3, and 4
JN3018	*Ex[gpa-10p::nls::YC2.60, lin-44p::GFP]; peIs2100[H20p::nls4::mCherry]*.	Figs. 1, 2, 3, and 4
JN3019	*Ex[gpa-13p::nls::YC2.60, lin-44p::GFP]; peIs2100[H20p::nls4::mCherry]*.	Figs. 1, 2, 3, and 4
JN3020	*Ex[gpc-1p::nls::YC2.60, lin-44p::GFP]; peIs2100[H20p::nls4::mCherry]*.	Figs. 1, 2, 3, and 4
JN3021	*Ex[lim-4p::nls::YC2.60, lin-44p::GFP]; peIs2100[H20p::nls4::mCherry]*.	Figs. 1, 2, 3, and 4
JN3022	*Ex[ins-1::nls::YC2.60, lin-44p::GFP]; peIs2100[H20p::nls4::mCherry]*.	Figs. 1, 2, 3, and 4
JN3023	*Ex[tdc-1::mTFP, lin-44p::GFP]; peIs2100[H20p::nls4::mCherry]*.	Figs. 1, 2, 3, and 4
JN3024	*Ex[ins-1(short)p::nls::YC2.60, lin-44p::GFP]; peIs2100[H20p::nls4::mCherry]*.	Figs. 1, 2, 3, and 4
JN3025	*Ex[mbr-1p::nls::YC2.60, lin-44p::GFP]; peIs2100[H20p::nls4::mCherry]*.	Figs. 1, 2, 3, and 4
JN3026	*Ex[nep-2sp::nls::YC2.60, lin-44p::GFP]; peIs2100[H20p::nls4::mCherry]*.	Figs. 1, 2, 3, and 4
JN3027	*Ex[npr-9p::nls::YC2.60, lin-44p::GFP]; peIs2100[H20p::nls4::mCherry]*.	Figs. 1, 2, 3, and 4
JN3028	*Ex[odr-2p::nls::YC2.60, lin-44p::GFP]; peIs2100[H20p::nls4::mCherry]*.	Figs. 1, 2, 3, and 4
JN3029	*Ex[sdf-9p::SDF9::GFP, lin-44p::GFP]; peIs2100[H20p::nls4::mCherry]*.	Figs. 1, 2, 3, and 4
JN3030	*Ex[sdf-9p::nls::GFP, lin-44p::GFP]; peIs2100[H20p::nls4::mCherry]*.	Figs. 1, 2, 3, and 4
JN3031	*Ex[ser-1p::nls::YC2.60, lin-44p::GFP]; peIs2100[H20p::nls4::mCherry]*.	Figs. 1, 2, 3, and 4
JN3032	*Ex[ser-2(prom2)p::mTFP, lin-44p::GFP]; peIs2100[H20p::nls4::mCherry]*.	Figs. 1, 2, 3, and 4
JN3033	*Ex[sra-6p::nls::YC2.60, lin-44p::GFP]; peIs2100[H20p::nls4::mCherry]*.	Figs. 1, 2, 3, and 4
JN2101	*Ex[tax-4p::nls::YC2.60, lin-44p::GFP]; peIs2100[H20p::nls4::mCherry]*.	Figs. 1, 2, 3, and 4
JN3035	*Ex[ttx-3p::nls::YC2.60, lin-44p::GFP]; peIs2100[H20p::nls4::mCherry]*.	Figs. 1, 2, 3, and 4
JN3036	*Ex[vem-1p::nls::YC2.60, lin-44p::GFP]; peIs2100[H20p::nls4::mCherry]*.	Figs. 1, 2, 3, and 4
JN3038	*qjIs11[glr-1p::svnls2::TagBFPsyn, ser-2(prom2)p::svnls2::TagBFPsyn]; peIs3042[eat-4p::svnls2::TagRFP675syn, lin-44p::GFP]; peIs2100[H20p::nls4::mCherry]; qjIs14[H20p::nls::YC2.60]*.	Figs. 3, 4, and S10
JN3039	*qjIs11[glr-1p::svnls2::TagBFPsyn, ser-2(prom2)p::svnls2::TagBFPsyn]; peIs3042[eat-4p::svnls2::TagRFP675syn, lin-44p::GFP]; peIs2100[H20p::nls4::mCherry]*.	Figs. 3, 4, and S10
JN3041	*Ex[ptr-10p::nls::YC2.60, lin-44p::GFP]; peIs2100[H20p::nls4::mCherry]*.	Fig. 1
ZIM945	*lite-1(xu-7); mzmIs4[Punc-31::NLSGCaMP5K; Punc-122::gfp]*	Figure S10
ZIM1048	*lite-1(ce314); mzmIs4[Punc-31::NLSGCaMP5K; Punc-122::gfp]*	Figure S10

### Brood size counting

The brood size was counted as previously described [35]. Briefly, an L4 animal was isolated to a seeded NGM plate and moved to a new seeded NGM plate every 24 h. Dead eggs in a plate were counted 48 h after the parent animal was moved into the plate. The age of worms (adult, L4, under L3) and male adults were counted 96 h after the parent worm was moved into the plate. The brood size was obtained as a sum of the number of dead eggs and the number of living animals. The adult ratio was obtained as the number of adult hermaphrodites divided by the brood size. The male ratio was obtained as the number of adult males divided by the brood size. The values from more than 8 biological replicates (*n* = 8 plates for N2, *n* = 9 plates for JN3038, *n* = 10 plates for JN3039, *n* = 11 plates for ZIM945, and *n* = 10 plates for ZIM 1048) were analyzed by Tukey’s post hoc test following one-way ANOVA (Kruskal-Wallis test) with N2 as a reference.

### Locomotion speed

The locomotion speed of adult animals was measured by using the worm tracking system that identifies and tracks tens of animals on a test plate [26]. Briefly, 30–50 adult animals were placed at the center of an 8.5-cm-diameter plate and images of the whole plate were acquired at 1 frame/s. The locomotion speed was obtained as the movement of running animals (i.e., not in the pause, reversal, or pirouette period) between 100 and 500 s. The median of locomotion speed was obtained from every plate, and the medians of 5 strains from 5 biological replicates were analyzed by Tukey’s post hoc test following one-way ANOVA (Kruskal-Wallis test) with N2 as a reference.

### Salt chemotaxis assay

The chemotaxis assays were performed as previously described [27] with slight modification. Briefly, Bristol N2 and JN3038 animals were raised on NGM plate with OP50 for 4 days and 6 days, respectively. The animals for conditioning with food were picked and moved to the conditioning plate including 25 mM or 100 mM NaCl with food and cultivated overnight. The animals for conditioning without food were exposed to conditioning liquid (5 mM potassium phosphate, pH 6.0, 1 mM CaCl_2_, and 1 mM MgSO_4_) with 25 mM or 100 mM NaCl for 1 h at room temperature [36]. The conditioned animals were placed on the center of an 8.5-cm-diameter plate with a NaCl gradient from 35 to 95 mM (assay plate). The animals were allowed to run for 30 min and chemotaxis index was calculated as (nA − nB)/(nTotal − nO), where nA, nB, and nO are the number of worms in area A (4-cm-diameter region with high salt), area B (4-cm-diameter region with low salt), and area O (maximum 2-cm-diameter region around the center), respectively. nTotal is the number of worms on the assay plate.

### Microscopy

A set of static 3D multi-channel images of *C. elegans* strains ranging from JN3000 to JN3036 and JN3041 was obtained as follows. Day 1 adult animals were stained by the fluorescent dye DiR (D12731, Thermo Fisher Scientific) with the standard method [24]. Briefly, the worms were incubated 2.5 h in M9 buffer with 10 μg/ml DiR, transferred to the NGM plate with food, and cultivated for 1 h. The stained animals were mounted on a 2% agar pad and paralyzed by sodium azide. The fluorescence of the fluorescent proteins and the dye was observed sequentially using laser scanning confocal microscopy (Leica SP5 with 63× water immersion lens and 2× zoom). The sizes of the images along the *x* and *y* axes were 512 and 256 voxels, respectively, and the size along the *z* axis varied depending on the diameter of the animal. The sizes of a voxel along the *x*, *y*, and *z* axes were 0.240, 0.240, and 0.252 μm, respectively. Other optical settings varied depending on the conditions of the sample and the microscopy. Typically, TagBFP was excited by a 405-nm laser and detected at 415–470 nm. YC2.60 was excited by a 458-nm laser and detected at 465–505 nm (for CFP) and 525–570 nm (for YFP) simultaneously. mCherry was excited by a 543-nm laser and detected at 570–620 nm. TagRFP675 and DiR were excited by a 633-nm laser and detected at 660–745 nm. Each voxel was scanned twice at 400 Hz.

A set of 3D multi-channel images of strain JN3039 was obtained as described above without using the fluorescent dye DiR.

A set of 3D multi-channel images of strain JN3038 was obtained as follows. Day 1 adult animals were conditioned on a NGM plate with OP50 [26]. The conditioned animals were introduced and held in a microfluidic device called olfactory chip [37]. The depth and width of the fluid channel in the chip were modified to 35 μm each in order to reduce the distortion of the worms. The animals and their head neurons moved to some extent in the device because the animals were not paralyzed. The fluorescence of the tagBFP, tagRFP675, and mCherry channels was observed simultaneously using customized spinning disk confocal microscopy. TagBFP was excited by a 405-nm laser and detected at 454–496 nm. mCherry was excited by a 561-nm laser and detected at 514–625 nm. TagRFP675 was excited by a 637-nm laser and detected at 754–816 nm. The exposure time was 500 ms. The sizes of the images along the *x*, *y*, and *z* axes were 512, 256, and 50 voxels, respectively. The sizes of a voxel along the *x*, *y*, and *z* axes were 0.28, 0.28, and about 0.77 μm, respectively. For obtaining a time series of 3D images of mCherry using JN3038, the exposure time was 4 ms, the size of the images along the *z* axis was 22 voxels, and the size of a voxel along the *z* axis was 2.00 μm.

### Image analysis for the neuron ID dataset

All the nuclei in the images were detected by our image analysis pipeline roiedit3D [10] and corrected manually. The cells stained by the chemical dye were identified as reported [24]. The cells marked by cell-specific promoters were identified based on the reported expression patterns [38, 39] and positions of the nuclei [3, 40]. The nuclei of the pharyngeal cells were also identified based on the positions of the nuclei [40]. The cells surrounded by the identified cells were also identified if possible.

### Correction of the posture of worms

First, all the positions of nuclei in a worm determined by roiedit3D were analyzed by PCA and the first principal component axis (PC1 axis) was defined as the anterior-posterior axis. The positions of the nuclei were fitted with a quadratic function along the PC1 axis (see Additional file 2: Figure S1). The determined quadratic function minimizes the sum of the squared distances from the fitted line to the positions of nuclei along PC2–PC3 axis. The positions were corrected so that the quadratic line was straightened and at the same time the rolled posture of the animal was corrected. The positions of the nuclei were projected onto the plane with PC2–PC3 axes, and the sparsest direction from the center was defined as the dorsal direction. The positions were rotated along the PC1 axis so that PC1 (antero-posterior axis) is aligned to the *x* axis and the dorsal direction is aligned to the positive direction of the *y* axis. Then, we estimated the anterior direction based on the density of the lateral cells. The densest position was set as the origin of the anterior-posterior axis (translation). The origins of the dorsal-ventral and left-right axes were the same as the origin of the PC2 and PC3 axes. The worms can be aligned by these procedures. The positions of the animals in the neuron ID dataset were corrected precisely based on the positions of the dye-stained cells.

### Variation of relative positions

Variation of the relative position of a cell pair was calculated as the determinant of the covariance of relative cell positions. Let *X*_*i*_ and *Y*_*i*_ be the positions of the cells *X* and *Y* in the *i*th animal, respectively, and the cells were identified in *n* animals.

$$ X=\left\{{X}_1,{X}_2,\dots, {X}_n\right\} $$

and

$$ Y=\left\{{Y}_1,{Y}_2,\dots, {Y}_n\right\} $$

are n-by-3 matrices of the positions of the cells *X* and *Y*, respectively. Then, the variation of relative positions of cell *X* and cell *Y* is

$$ V\left(X,Y\right)=\det \left(\operatorname{cov}\left(X-Y\right)\right). $$

For visualization, *V*(*X*, *Y*) was divided by the median value of all *V*. The pairs with *n* ≤ 3 were ignored because the determinant of covariance cannot be calculated.

Less varying cell pairs were found based on permutation of animals (permutation test). A permutation of the vector *X* permutes the order of elements of the vector *X*, for example,

$$ \mathrm{perm}(X)=\left\{{X}_j,\dots, {X}_1,\dots, {X}_k\right\}. $$

The pair of cells *X* and *Y* was regarded as less varying if

$$ V\left(X,Y\right)\le {}^{\forall }V\left(\mathrm{perm}(X),Y\right). $$

For the pairs of 4 ≤ *n* ≤ 10, all the permutations (equal to or less than 10 ! ~3.6 × 10^6^ combinations) were calculated. For the pairs of *n* > 10, 1 × 10^7^ permutations were randomly selected and calculated.

### Stability of expression

The positive ratio is a ratio of the number of positive (promoter-expressing) cells over the total number of the same cells (= number of tested animals). Let *n*_*X*_ be the number of cells that express the landmark fluorescent protein driven by a promoter and let *m* be the number of tested animals for the promoter. The positive ratio *p*_*X*_ is

$$ {p}_X=\frac{n_X}{m}. $$

Stability of expression was calculated as an average of the positive ratio over the cells in which at least one cell is positive for the promoter. Let *l* be the number of cells in which the promoter drives expression of the landmark fluorescent protein in an animal. The stability of expression *s* is

$$ s=\frac{1}{l}{\sum}_{X=1}^l{p}_X. $$

### The algorithm for searching optimal combination of cell-specific promoters and the definition of the sparseness

The most important factor for selecting promoters in order to improve annotation accuracy is to achieve a checkerboard-like coloring pattern for the ease of distinguishing neighboring cells. A simple metric to account for this factor is to sum the number of neighboring cell pairs that exhibit a different color based on cell-specific promoters, where each pair is inversely weighted by the distance between the two neurons. Such a metric can be considered as a modification to an Ising model in physics. We choose a Gaussian probability model for the weighting function with an empirically chosen value of the standard deviation to be 9.6 μm. The metric *M* can be written as

$$ M=\frac{1}{2}\sum \limits_{X\in S}\sum \limits_{Y\in S}I\left({L}_X,{L}_Y\right)w\left(X,Y\right) $$

$$ I\left({L}_X,{L}_Y\right)=\left\{\begin{array}{c}1\ \mathrm{if}\ {L}_X\ne {L}_Y\\ {}0\ \mathrm{if}\ {L}_X={L}_Y\end{array}\right. $$

$$ w\left(X,Y\right)=\mathrm{N}\left(X|Y,9.6\right), $$

where *S* is a set of all cells in an animal. *X* and *Y* are positions of cell X and cell Y, respectively. *L*_*X*_ is the label of cell *X* and *L*_*X*_ = (1, 0) means that landmark protein of color 1 is expressed in the cell *X* but that of color 2 is not expressed. Because the experimental setup has a limited amount of channels, we are able to perform an exhaustive search for all possible combinations of the available promoters, and compare the final values of the metric as a reference for choosing the combination of cell-specific promoters used in our experiment. We evaluated all the combinations for 3 promoters and 2 colors (20,825 combinations). The scores of the single promoter for single color were used as the index of sparseness.

### Generating atlases

To obtain an atlas with fully annotated cells, we need to combine positional information of cells from multiple partially annotated images while maintaining the relative position between the cells as much as possible. We achieve this goal by maximizing the consistency (or smoothness) of a displacement flow when combining different images, for which the displacement flow is defined as follows.

Suppose that in two images, denoted by *I*_0_ and *I*_1_, there coexist *C* annotated cells. The displacement of cell *i* is denoted by ${\boldsymbol{d}}_i={\boldsymbol{x}}_i^1-{\boldsymbol{x}}_i^0$ where ${\boldsymbol{x}}_i^0$ and ${\boldsymbol{x}}_i^1$ denote the positions of the cell in *I*_0_ and *I*_1_, respectively. Then, we define a displacement flow field ***d***_0 → 1_(***x***) from *I*_0_ to *I*_1_ on the entire space ***x*** ∈ *ℝ*^3^:

1 $$ {\boldsymbol{d}}_{\mathbf{0}\to \mathbf{1}}\left(\boldsymbol{x}\right)=\frac{\sum_{\boldsymbol{i}=\mathbf{1}}^{\boldsymbol{C}}\boldsymbol{N}\left(\boldsymbol{x}|{\boldsymbol{x}}_{\boldsymbol{i}}^{\mathbf{0}},\boldsymbol{\Sigma} \right){\boldsymbol{d}}_{\boldsymbol{i}}}{\sum_{\boldsymbol{i}=\mathbf{1}}^{\boldsymbol{C}}\boldsymbol{N}\left(\boldsymbol{x}|{\boldsymbol{x}}_{\boldsymbol{i}}^{\mathbf{0}},\boldsymbol{\Sigma} \right)}. $$

Here, *N*(***x***| ***μ***, *Σ*) denotes the density function of the normal distribution with mean ***μ*** and covariance Σ (please note that *Σ* is 3 × 3 covariance matrix and determines the effective range of the displacement of a cell). This represents a flow field function interpolated by the given displacements of the *C* cells in the two images. When taking the weighted average in the calculation of ***d***_0 → 1_(***x***), larger weights are assigned to the displacements of more neighboring cells with respect to ***x*** in *I*_0_.

To generate an atlas, we conducted the following steps (see Fig. 4a):

1. Set a randomly ordered sequence {*I*_1_, …, *I*_311_} of the 311 partially annotated animals. We discard the sequence if the *I*_1_ has less than 60 annotated cells.
2. For *t* ∈ {2…, 311}, cells in *I*_*t*_ were sequentially aligned to those in *I*_1_ as follows:
  A) The positions of all annotated cells in *I*_1_ were unchanged.
  B) All annotated cells that coexisted in both *I*_1_ and *I*_*t*_ were used to calculate the displacement field ***d***_*t* → 1_(***x***) with a pre-determined *Σ* (Eq. 1).
  C) All cells annotated in *I*_*t*_ but not in *I*_1_ with their positions denoted by ***x***_*t*_ were shifted and aligned to *I*_1_ according to ***x***_1_ ← ***x***_*t*_ + ***d***_*t* → 1_(***x***_*t*_). Add them to the annotated cells in *I*_1_.
  D) Terminate the iteration if all annotated cells have been aligned in the synthesized reference image.

In this scheme, a spatial pattern of produced cells was largely affected by the interpolated flow fields. In general, the performance will be poor if the number of observed source displacements was small. To reduce such instability, we skipped *I*_*t*_ and used it later when the *I*_*t*_ shared less than half cells annotated in common with *I*_1_. Repeating this procedure, we generated 3000 reference samples.

The generated reference samples serve as a set of virtual atlases that imitate observed topological variations of cellular positions across different worm samples. To obtain more realistic atlases, we optimized *Σ* = diag(*σ*_1_, *σ*_2_, *σ*_3_) in Eq. 1, which is the parameter to control the smoothness of displacements in the sequential alignments. We defined an objective function to reflect the similarity of the topological variations between our neuron ID data set and the generated atlas. By optimizing such objective function and taking the optimal values of the parameters as a reference, we selected an empirical value of *Σ* = diag(9.6 μm, 9.6 μm, 9.6 μm). Details of the objective function and optimization are in Additional file 27: Note S1.

### Bipartite graph matching

Detected cells in a target animal and an atlas were matched using the Hungarian algorithm to solve the bipartite graph matching problem. The matching was achieved by comparing one or more selected features between cells. Here, features refer to some quantitative properties for the cells that can be used to distinguish the identity of a cell from another. The most fundamental feature is the positions of cells. Other typical features include cell volume, fluorescence intensities, and manual annotation results. For performance measurements, positions and expression of landmark proteins (i.e., binarized fluorescent intensities) were used. Manual annotation results can be used as an additional feature for improving the automatic estimations with feedback from human annotation, but were not considered in performance test except for Fig. 4f. With such features, the dissimilarity of cells was represented by a matrix *A*, where the {*i*, *j*} entry is the distance of the feature values between the *i*th cell in the target and the *j*th cell in the atlas. When there are *N*_*f*_ features chosen, we can assemble them into a single matrix *A*_BGM_ through a weighted sum:

$$ {A}_{\mathrm{BGM}}={\sum}_{n=1}^{N_f}{w}_n{A}_n, $$

where *w*_*n*_ is the weight for each feature. We use *w*_distance_ = 1, *w*_landmark_ = 20. For feedback from human annotation, the assignments incompatible with the human annotation have infinity dissimilarity. With a given assignment, we can calculate the sum of the dissimilarity values in *A*_BGM_ that correspond to the selected matching. A modified Hungarian algorithm [41] was used to minimize the total distance with respect to all possible assignments under the constraint of one-to-one matching.

### Majority voting

Multiple name assignments of a cell in the subjective animal were obtained by repeating the bipartite graph matching using 500 different atlases. Each assignment was considered as one vote, and the estimated names for a target cell were ranked by vote counts. The estimation for a cell was independent of each other, and multiple cells may have the same estimated names. If the non-overlapping result is required, one can assemble cost matrix based on vote counts and apply the Hungarian algorithm.

### Calculation of error rate of automatic annotation

All the detected cells in a target animal other than hypodermal cells were used as a target. The names of the cells were estimated by our automatic annotation method based on their positions. The expression of landmark promoters were also used for Fig. 4d and Additional file 22: Figure S15. The estimated results are compared to the human annotation (ground truth). The error rate was calculated as 1 – (*N*_correct_)/(*N*_annotated_) for each animal, where *N*_annotated_ is the number of human-annotated cells (ground truth) and *N*_correct_ is the number of cells whose annotation is correctly estimated. Our automatic annotation method returns multiple ranked candidates for a target cell. The rank *R* error rate indicates that it is considered correct if the correct annotation appeared in the top *R* estimations. Un-annotated cells were ignored in calculating the error rate. The animals that have less annotated cells were removed to avoid the effect of deviation of the annotated cells.

## Supplementary information

**Additional file 1: Table S1.** Summary of neuron ID dataset including expression patterns of the promoters.

**Additional file 2: Figure S1.** Correction of posture of the worms. (A) An example bright-field image of the head region of an adult animal with curved posture. (B) The positions of the cells in the animal (shown as blue circles) are projected onto the plane with PC1-PC2 axes and the plane with PC2-PC3 axes, where PC1 is the 1st principal component (see Methods). The fitted quadratic curve is shown as the red line. (C) The corrected position of the cells.

**Additional file 3: Figure S2.** Performance and robustness of the posture correction. (A) The variation of cell positions was evaluated as the volume of ellipsoid (corresponding to the determinant of covariance of cell positions; see Fig. 2) before and after the posture correction. The variation of cell positions was plotted against the mean cell position in the anterior-posterior (AP) axis (circles and crosses). Note that the y axis is in logarithmic scale. The lines indicate moving average obtained with a window of ±15 μm. (B) The moving average of variation of cell positions after posture correction steps that consist of principal component analysis (PCA), quadratic curve fitting, rotation, and translation (see Methods), each of which reduced the variations. (C) Up to 20% of the cells were randomly removed before the posture correction that simulates overlooking of cells in the nucleus detection step. The lines indicate the moving averages of variation of cell positions after the posture correction. The lines are overlapped, suggesting that the posture correction step is robust for the overlooking of cells. (D) Up to 20% of the cells either in the anterior side or in the posterior side were removed before the posture correction that simulates cells moving out of the view of the images. The side in which the cells were removed was randomly chosen in each animal. The lines indicate the moving averages of variation of cell positions after the posture correction. The lines are overlapped, suggesting that the posture correction step is robust for the movement of the cells. The summary statistics of (A)-(D) are in Additional file 4: Table S2. (E) The cell counts are shown against the mean cell position in the AP axis. The small counts of the cells in the posterior side might increase the instability of the moving averages of ellipsoid volumes in the posterior side. (F) The variation of cell positions is shown against the cell counts. The variation and the cell counts did not seem to correlate in general.

**Additional file 4: Table S2.** Summary statistics for Additional file 3: Figure S2.

**Additional file 5: Figure S3.** Movements of the cells during time-lapse imaging. (A) The mean position and covariance of cell positions before the translation correction are shown as ellipsoids (see Fig. 2). An adult animal of JN3038 strain (see below) was introduced in the customized olfactory chip (see Methods) and imaged for about 20 min (6000 volumes). The nuclei in the volumetric movie were detected and tracked. Note that the origins of the axes are the same as those of the obtained raw images and the cell positions cannot be compared directly to other data including Fig. 2a. (B) The mean position and covariance of cell positions after the translation correction are shown as ellipsoids. The volumes of ellipsoids are smaller than that in Fig. 2a, indicating that the temporal movements alone cannot explain the large variations of cell positions shown in Fig. 2. The summary statistics are in Additional file 6: Table S3.

**Additional file 6: Table S3.** Summary statistics for Additional file 5: Figure S3.

**Additional file 7: Figure S4.** Overlay plot of cell positions for all worms. (A) The positions of cells in the left half of the body for all worms are plotted. Colored circles indicate the positions of identified cells. Gray circles indicate the positions of unidentified cells. Different colors mean different identities. (B) Same as (A) but only for identified cells. (C) Same as (A) but only for identified non-pharyngeal cells. (D) Same as (A) but only for identified pharyngeal cells.

**Additional file 8: Figure S5.** Specific-cell-centered landscape. (A) Original landscape as a reference. This panel is basically the same as Fig. 2a, but several cells are removed for visibility. (B) ASKR-centered landscape. The position of ASKR cell is indicated as a cross. (C) MI-centered landscape. The position of MI cell is indicated as a cross. The same cell has the same color in (A)-(C). The cells in the right side are shown. Several cells are removed for visibility.

**Additional file 9: Figure S6.** Less varying neuron pairs. (A) Less varying neuron pairs were obtained by random permutation of animals (see Methods) and the less varying pairs in the left half of the body are shown by red lines. The pairs including pharyngeal cells were omitted for visualization. (B) The less varying pairs including pharyngeal cells are shown by red lines.

**Additional file 10: Figure S7.** Position of posterior pharyngeal bulb affects cell positions. (A-C) A/P (A), D/V (B) and L/R (C) positions of dye positive cells plotted against relative A/P positions of pharynx. The relative A/P positions of pharynx were calculated from the mean difference of positions of pharyngeal cells from reference. Blue crosses indicate the cell positions in respective animals. The red lines and the red dotted lines indicate regression lines and 95% confidence bounds, respectively. The summary statistics are in Additional file 11: Table S4.

**Additional file 11: Table S4.** Summary statistics for Additional file 10: Figure S7.

**Additional file 12: Figure S8.** Stability and sparseness of expression pattern. (A) Number of positive cells and stability of expression of the cell-specific promoters. Same as Fig. 3a but fully labeled. (B) Number of positive cells and sparseness of expression pattern of the cell-specific promoters. Same as Fig. 3b but fully labeled.

**Additional file 13: Table S5.** Evaluation result of promoter combinations.

**Additional file 14: Figure S9.** An example fluorescent image of JN3039 strain and annotated cell names. Zoomed version of Fig. 3d-e for visibility.

**Additional file 15: Dataset S2.** Positions of nuclei and expression patterns of landmark fluorescence in the whole-brain imaging strains as the test data for automatic annotation and corresponding static 3D images.

**Additional file 16: Figure S10.** Health of 4D strains. (A) Brood size of 4D strains including JN3038, JN3039, ZIM945 and ZIM 1048 were compared to N2. The animals were counted at 96 h after the parent animal was put on the plate (see Methods). The medians and the standard errors are shown. The sample size are *n* = 8 plates for N2, *n* = 9 plates for JN3038, *n* = 10 plates for JN3039, *n* = 11 plates for ZIM945, and n = 10 plates for ZIM 1048. The *p*-values of Tukey’s post hoc test following one-way ANOVA (Kruskal-Wallis test) to N2 are shown. The summary statistics are in Additional file 17: Table S6. (B) The ratio of adult hermaphrodite in (A). (C) The ratio of adult male in (A). (D) The locomotion speed of adult animals (see Methods). The means and the standard errors for *n* = 5 assays are shown. The p-values of Tukey’s post hoc test following one-way ANOVA (Kruskal-Wallis test) to N2 are shown. (E) Chemotaxis assay of JN3038. N2 and JN3038 animals were cultivated at 25 mM or 100 mM of NaCl with or without food. The conditioned animals were placed in the center of the assay plate with a NaCl gradient from 35 to 95 mM. The animals migrate to either side of the plate and chemotaxis index was calculated. A high chemotaxis index means the worms migrated to a high salt region. Mean ± SEM for *n* = 6 assays are shown. The p-values were obtained from a statistical test between JN3038 and N2 at the same condition (Wilcoxon rank sum test).

**Additional file 17: Table S6.** Summary statistics for Additional file 16: Figure S10.

**Additional file 18: Figure S11.** Comparison of the synthetic atlas and the neuron ID dataset. (A) Visualization of the variation of the cell positions in synthetic atlas. The cells and colors are the same as Fig. 2a. (B) Distance of mean position of cells between the atlas and the neuron ID dataset. (C) Ratio of volume of ellipsoid (covariance of the positions of the cell) between the atlas and the neuron ID dataset. (D) Comparing the variation of relative positions of dataset and that of atlas. The color indicates how many times the neuron pair is co-detected in an animal of the neuron ID dataset.

**Additional file 19: Figure S12.** Variation of relative position of cell pairs. Variation of relative position of cell pairs. Orders of cells and colors are the same as in Fig. 2c.

**Additional file 20: Figure S13.** Error rate of each bipartite matching and majority voting. Error rate of each bipartite matching and majority voting are shown in the blue histogram and the black lines, respectively. The names of the cells were estimated based on their positions. The error rate was calculated as 1 – (*N*_*correct*_)/(*N*_*annotated*_) for each animal, where *N*_*annotated*_ is the number of human-annotated cells (ground truth) and *N*_*correct*_ is the number of cells whose annotation by the algorithm was correct. Cells un-annotated by human were not included in the calculation of error rate. The rank R indicates that it was considered correct if the correct annotation appeared in the top R estimations by the algorithm.

**Additional file 21: Figure S14.** Relationship between error rate of automatic annotation for JN3039 and detected count in the neuron ID dataset. The error rate of automatic annotation for cells identified in JN3039 and detected count of the cell in the neuron ID are shown. The red lines and the red dotted lines indicate regression lines and 95% confidence bounds, respectively. The slope is − 4.55e-4 and the confidence interval is from − 0.0659 to 0.0169. The p-value is 0.0247.

**Additional file 22: Figure S15.** Error rates of the automatic annotation method for the animals in a microfluidic chip. Error rates of the automatic annotation method for the animals in a microfluidic chip for whole-brain activity imaging (JN3038 strain). Mean ± standard deviation over 12 animals are shown.

**Additional file 23: Dataset S3.** A tutorial for semi-automatic annotation using our software.

**Additional file 24: Figure S16.** Correct rate of automatic annotation and its improvement by manual annotation. (A) The effect of prior manual annotation on the correct rate of automatic annotation. The annotation of a single cell type was specified manually before performing the automatic annotation. The error rates of automatic annotation for cells identified in JN3039 are shown. (B) Improvement of correct rate was obtained by subtracting the original correction rate from the correction rate with prior manual annotation.

**Additional file 25: Figure S17.** Most valuable cells for improving accuracy of automatic annotation. (A) Improvement of correct rate for prior annotated cell itself. Difference of correct rate is 0.9 when the correct rate of the cell in original case (i.e. without prior annotation) is 0.1. (B) Mean improvement of correct rate for the cells other than the prior annotated cells. Prior annotation of SMDVL achieved about 0.59% improvement of correct rate, indicating that 1.1 cells were corrected in addition to SMDVL. (C) Mean improvement of correct rate through all cells. Prior annotation of SMDVL achieved about 1.1% improvement of correct rate, indicating that 2.0 cells were corrected.

**Additional file 26: Dataset S4.** Sequences of the promoters in Table 1.

**Additional file 27: Note S1.** Optimization of parameters for atlas generation.

**Additional file 28: Dataset S5.** All codes for the GUI RoiEdit3D and analysis pipeline to make figures.

## Data Availability

All data generated or analyzed during this study are included in this published article, its supplementary information files, and publicly available repositories. The datasets supporting the conclusions of this article, including Dataset S1, are available from Figshare (10.6084/m9.figshare.8341088) [23].
