# Supplementary figures and images for: Neuron ID dataset facilitates neuronal annotation for whole-brain activity imaging of C. elegans
Source: BMC Biol. 2020 Mar 19;18:30. doi: 10.1186/s12915-020-0745-2 (PMC7081613; doi:10.1186/s12915-020-0745-2)

A

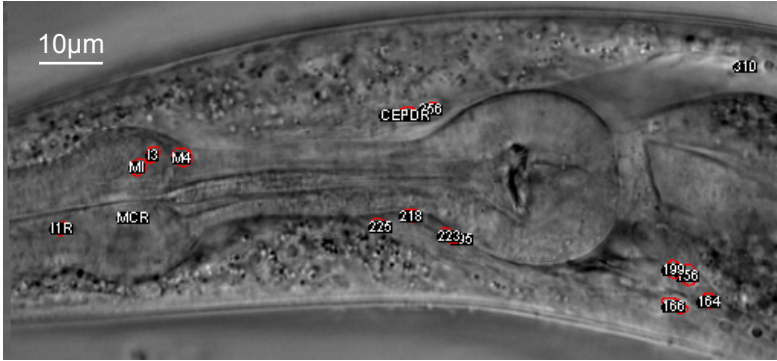

B

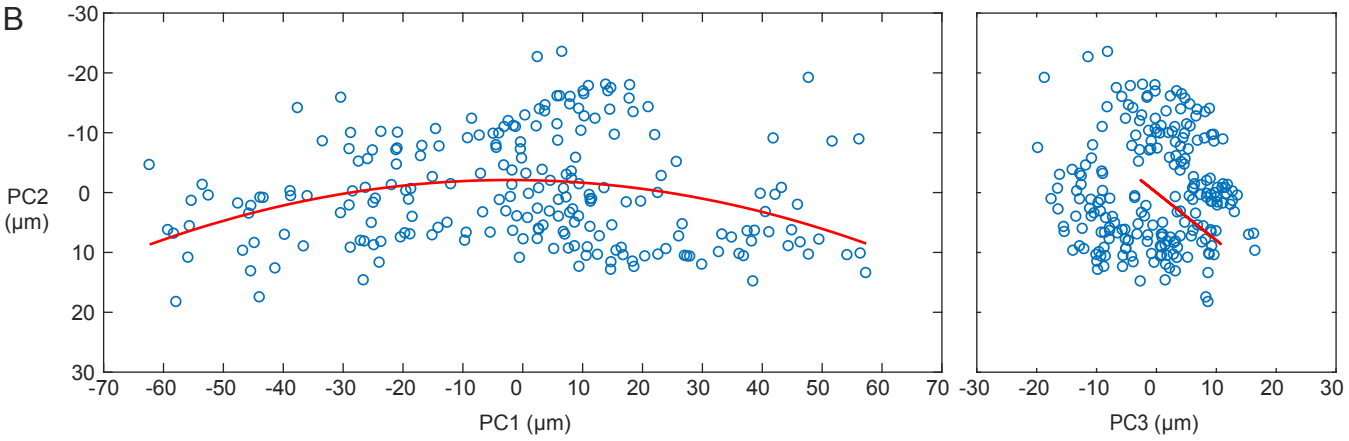

C

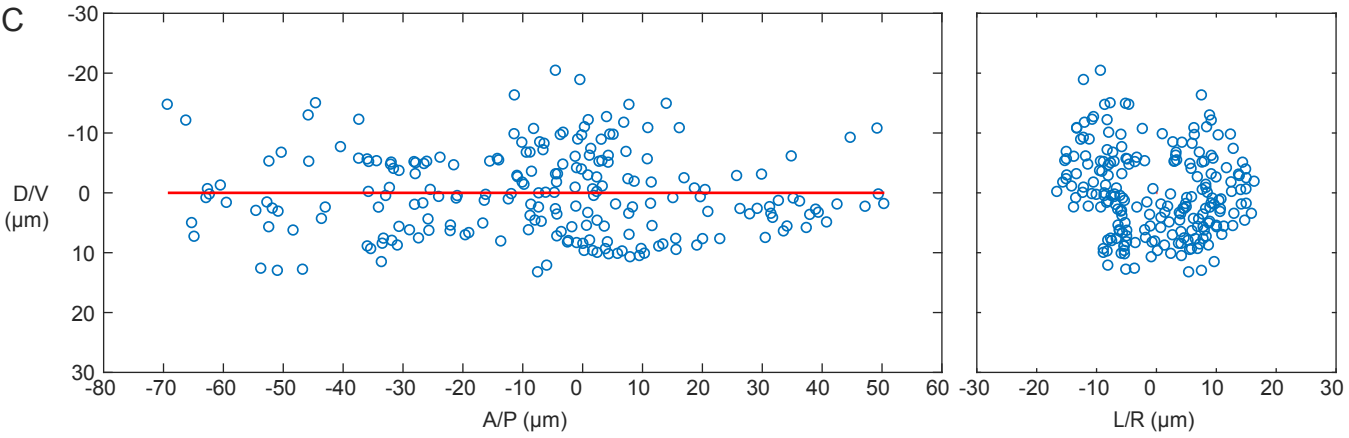

Supplement: Supplementary file 2 — Additional file 2: Figure S1. Correction of posture of the worms. (A) An example bright-field image of the head region of an adult animal with curved posture. (B) The positions of the cells in the animal (shown as blue circles) are projected onto the plane with PC1-PC2 axes and the plane with PC2-PC3 axes, where PC1 is the 1st principal component (see Methods). The fitted quadratic curve is shown as the red line. (C) The corrected position of the cells. [file 12915_2020_745_MOESM2_ESM.pdf]

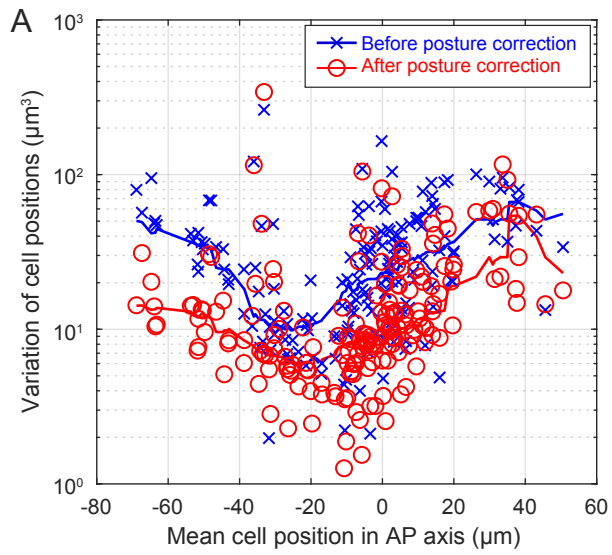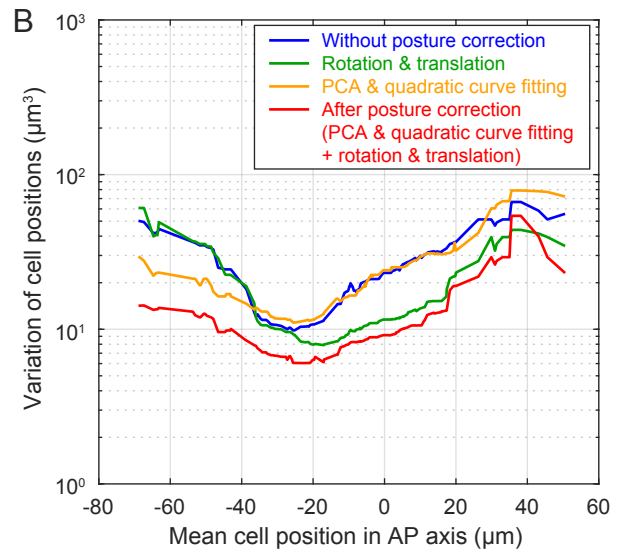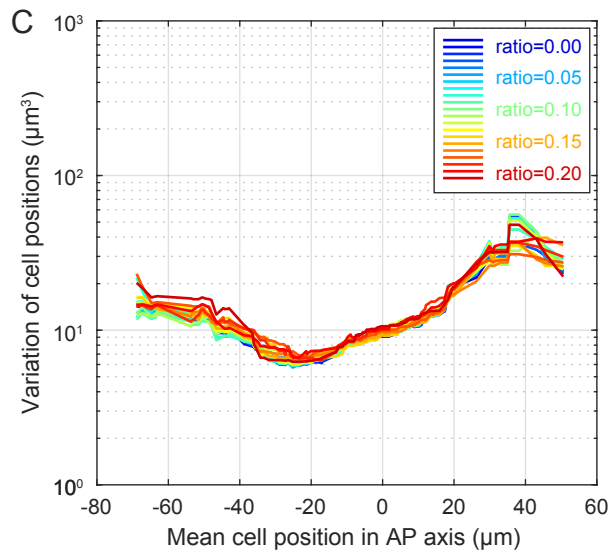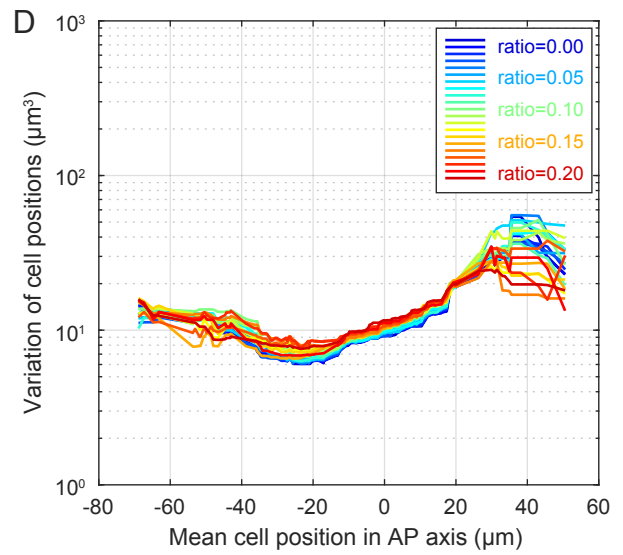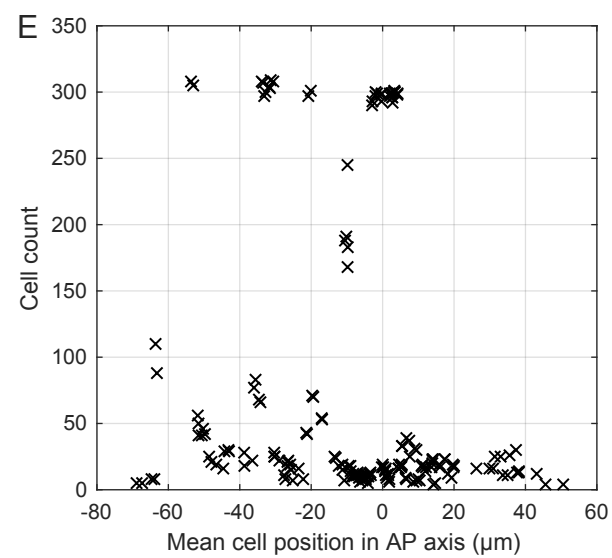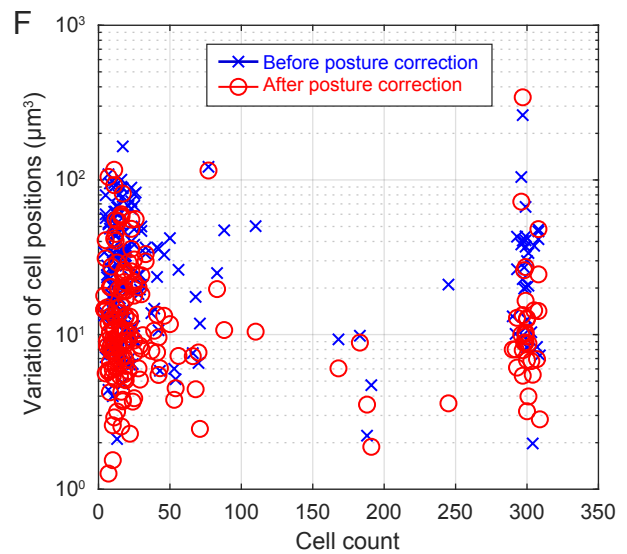

Supplement: Supplementary file 3 — Additional file 3: Figure S2. Performance and robustness of the posture correction. (A) The variation of cell positions was evaluated as the volume of ellipsoid (corresponding to the determinant of covariance of cell positions; see Fig. 2) before and after the posture correction. The variation of cell positions was plotted against the mean cell position in the anterior-posterior (AP) axis (circles and crosses). Note that the y axis is in logarithmic scale. The lines indicate moving average obtained with a window of ±15 μm. (B) The moving average of variation of cell positions after posture correction steps that consist of principal component analysis (PCA), quadratic curve fitting, rotation, and translation (see Methods), each of which reduced the variations. (C) Up to 20% of the cells were randomly removed before the posture correction that simulates overlooking of cells in the nucleus detection step. The lines indicate the moving averages of variation of cell positions after the posture correction. The lines are overlapped, suggesting that the posture correction step is robust for the overlooking of cells. (D) Up to 20% of the cells either in the anterior side or in the posterior side were removed before the posture correction that simulates cells moving out of the view of the images. The side in which the cells were removed was randomly chosen in each animal. The lines indicate the moving averages of variation of cell positions after the posture correction. The lines are overlapped, suggesting that the posture correction step is robust for the movement of the cells. The summary statistics of (A)-(D) are in Additional file 4: Table S2. (E) The cell counts are shown against the mean cell position in the AP axis. The small counts of the cells in the posterior side might increase the instability of the moving averages of ellipsoid volumes in the posterior side. (F) The variation of cell positions is shown against the cell counts. The variation and the cell counts did [file 12915_2020_745_MOESM3_ESM.pdf]

A

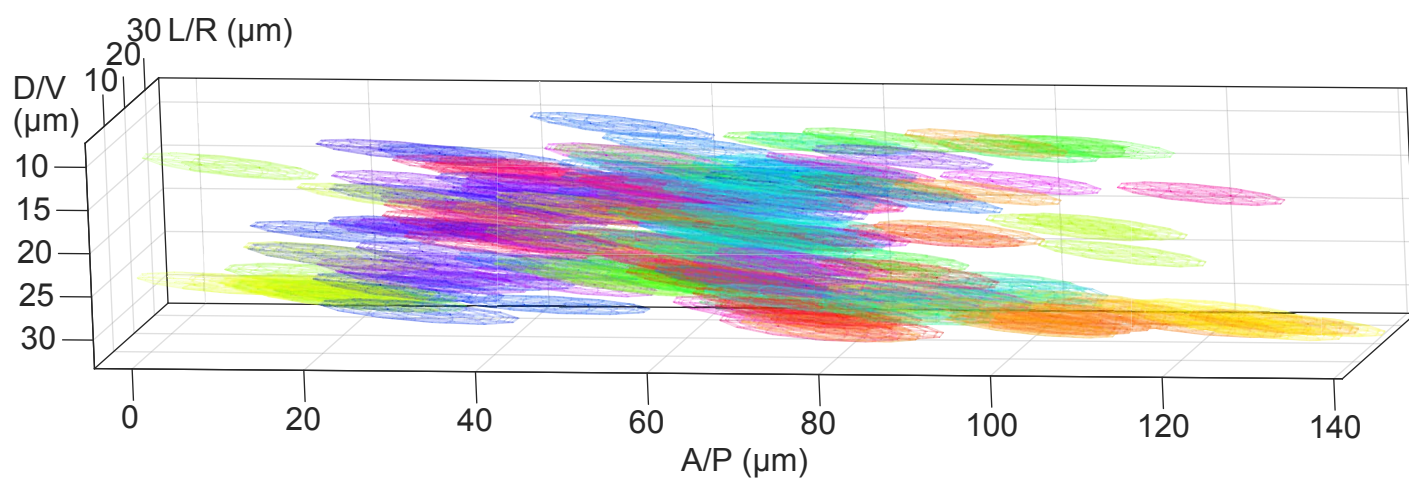

B

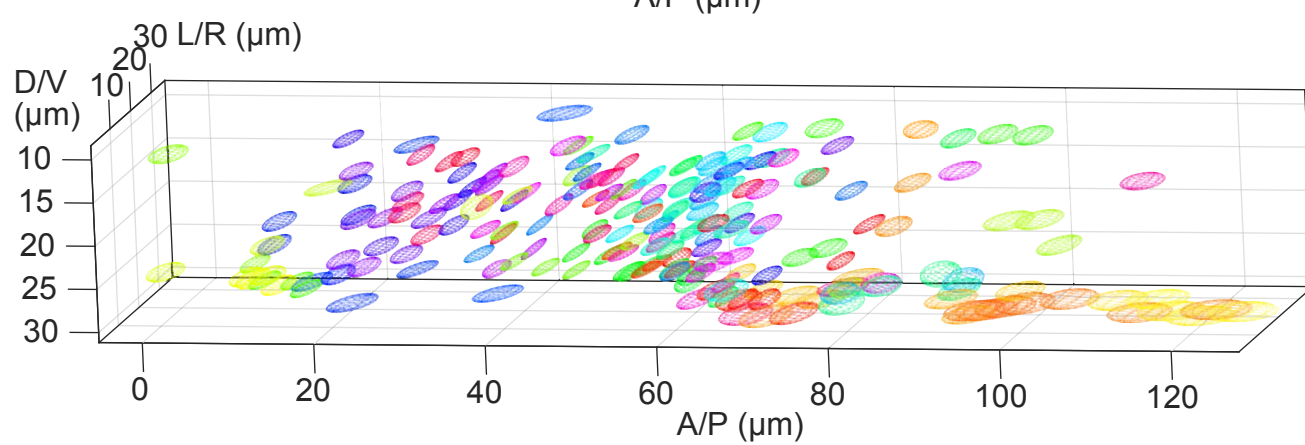

Supplement: Supplementary file 5 — Additional file 5: Figure S3. Movements of the cells during time-lapse imaging. (A) The mean position and covariance of cell positions before the translation correction are shown as ellipsoids (see Fig. 2). An adult animal of JN3038 strain (see below) was introduced in the customized olfactory chip (see Methods) and imaged for about 20 min (6000 volumes). The nuclei in the volumetric movie were detected and tracked. Note that the origins of the axes are the same as those of the obtained raw images and the cell positions cannot be compared directly to other data including Fig. 2a. (B) The mean position and covariance of cell positions after the translation correction are shown as ellipsoids. The volumes of ellipsoids are smaller than that in Fig. 2a, indicating that the temporal movements alone cannot explain the large variations of cell positions shown in Fig. 2. The summary statistics are in Additional file 6: Table S3. [file 12915_2020_745_MOESM5_ESM.pdf]

**A**

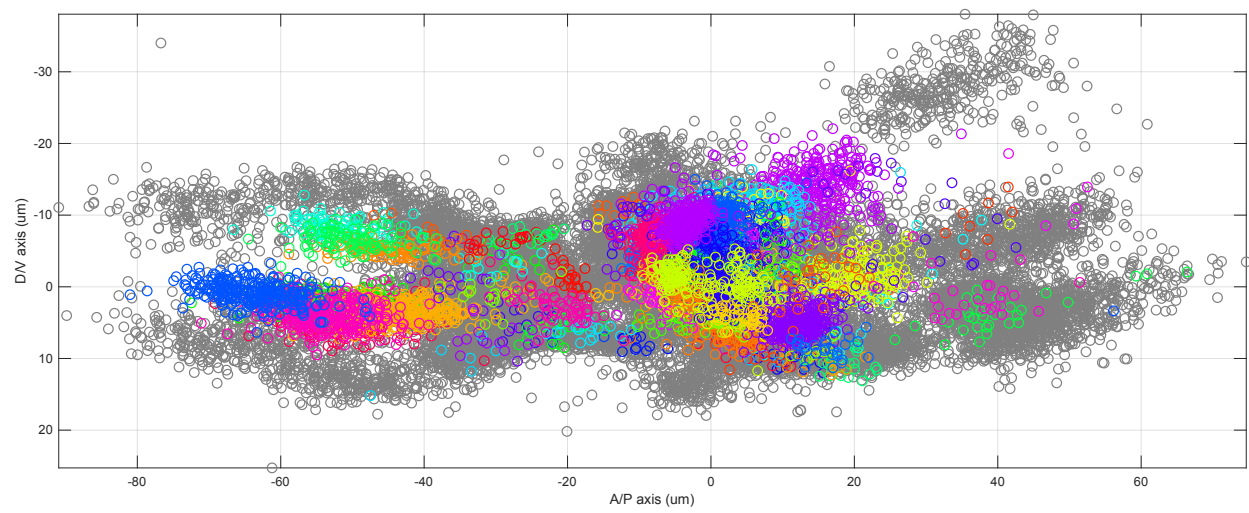

**B**

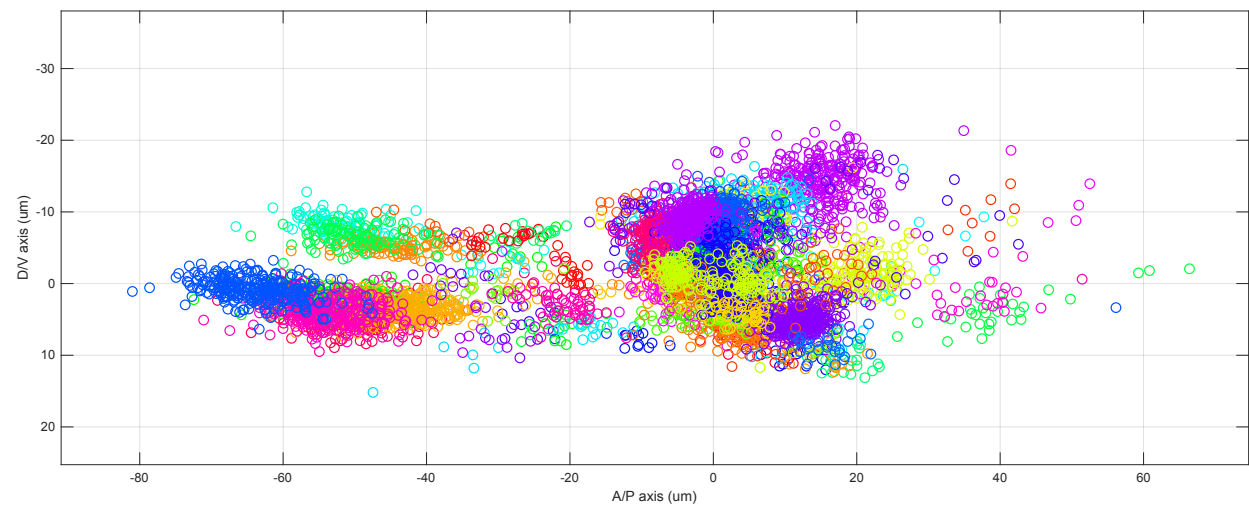

**C**

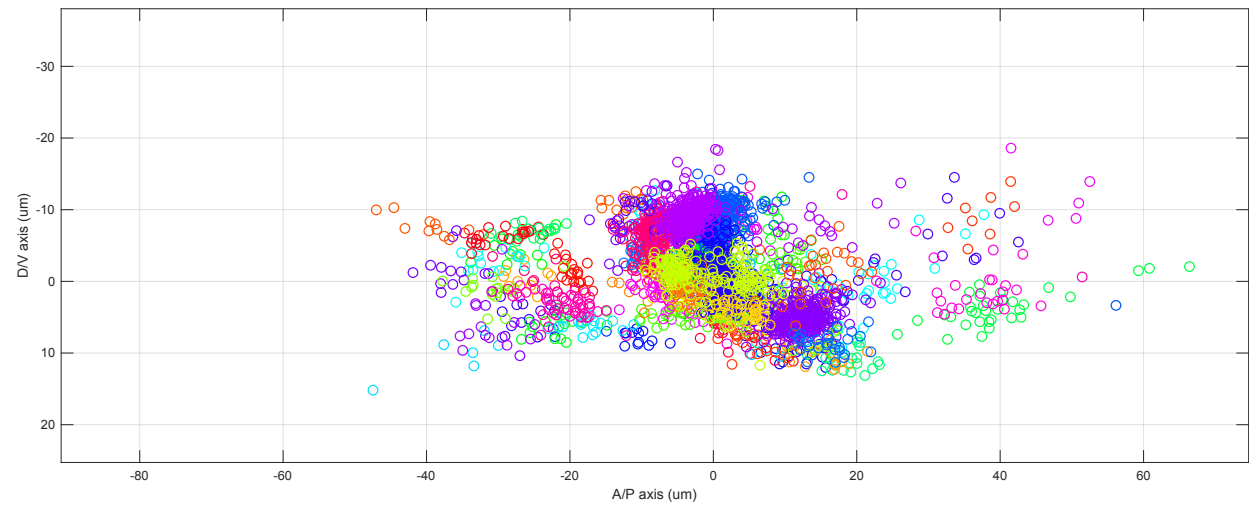

**D**

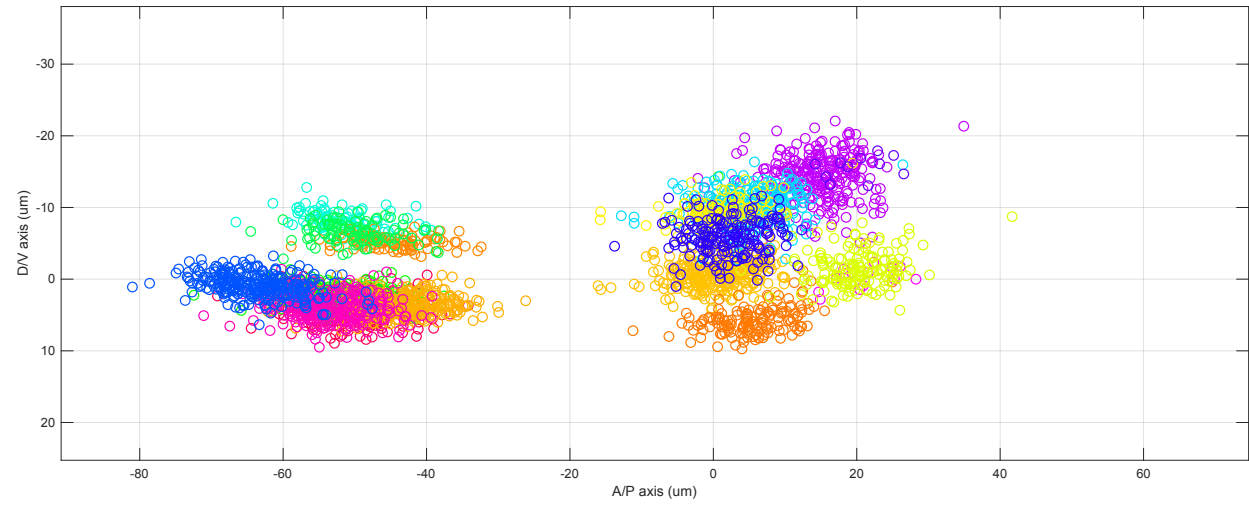

Supplement: Supplementary file 7 — Additional file 7: Figure S4. Overlay plot of cell positions for all worms. (A) The positions of cells in the left half of the body for all worms are plotted. Colored circles indicate the positions of identified cells. Gray circles indicate the positions of unidentified cells. Different colors mean different identities. (B) Same as (A) but only for identified cells. (C) Same as (A) but only for identified non-pharyngeal cells. (D) Same as (A) but only for identified pharyngeal cells. [file 12915_2020_745_MOESM7_ESM.pdf]

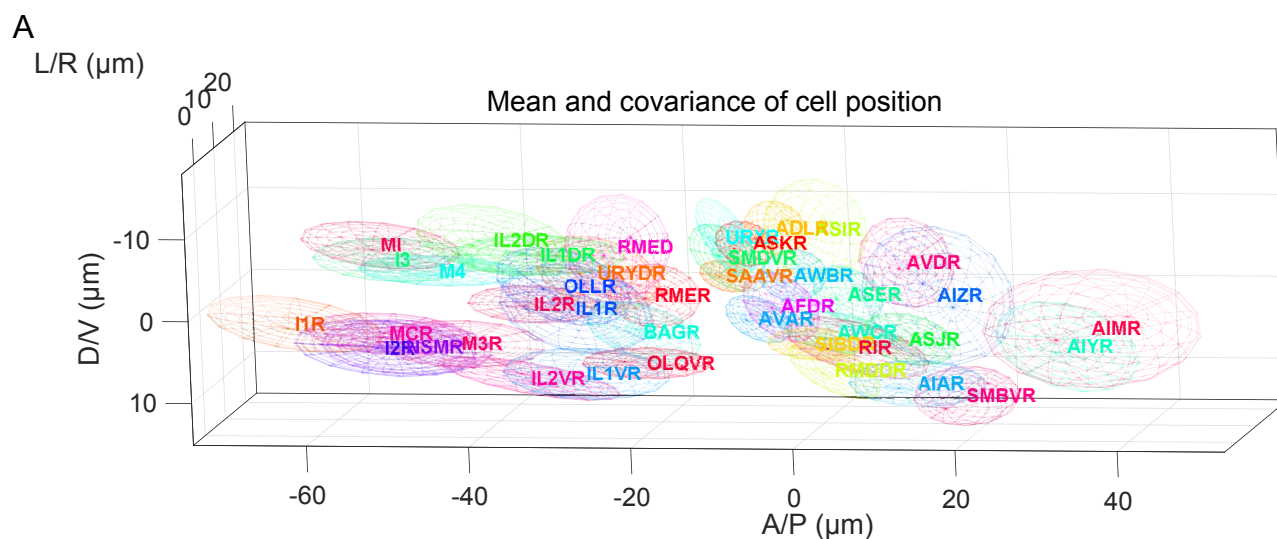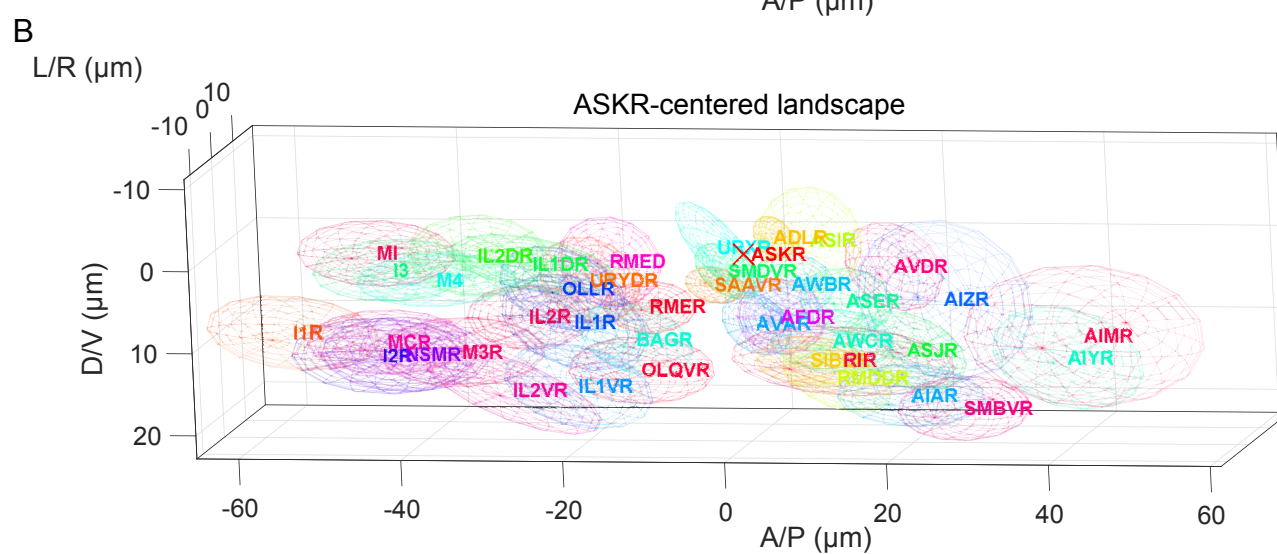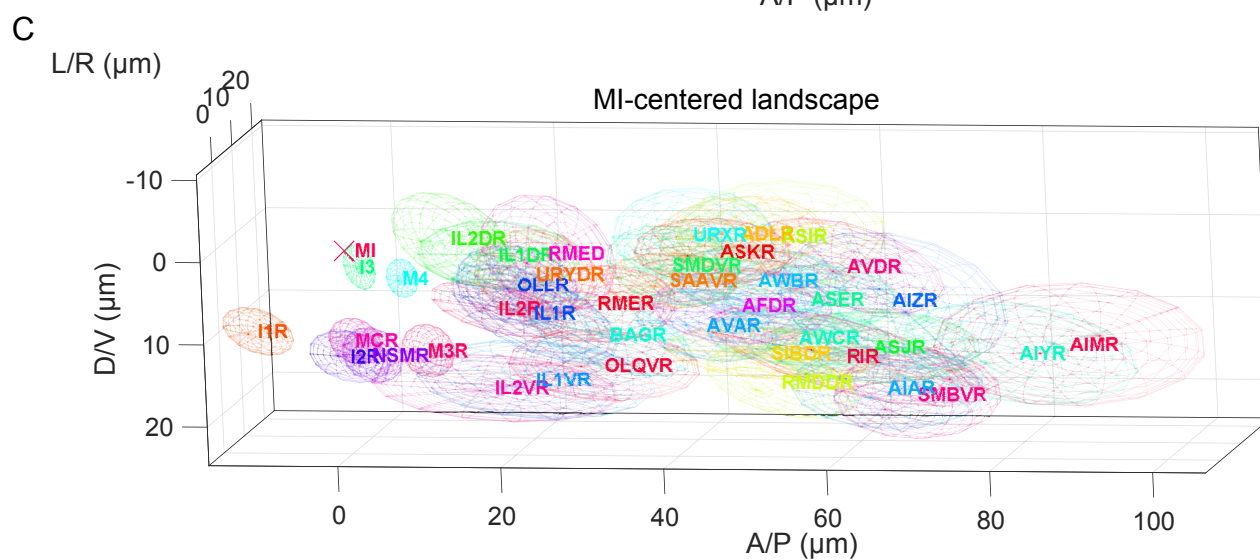

Supplement: Supplementary file 8 — Additional file 8: Figure S5. Specific-cell-centered landscape. (A) Original landscape as a reference. This panel is basically the same as Fig. 2a, but several cells are removed for visibility. (B) ASKR-centered landscape. The position of ASKR cell is indicated as a cross. (C) MI-centered landscape. The position of MI cell is indicated as a cross. The same cell has the same color in (A)-(C). The cells in the right side are shown. Several cells are removed for visibility. [file 12915_2020_745_MOESM8_ESM.pdf]

A: A/P position

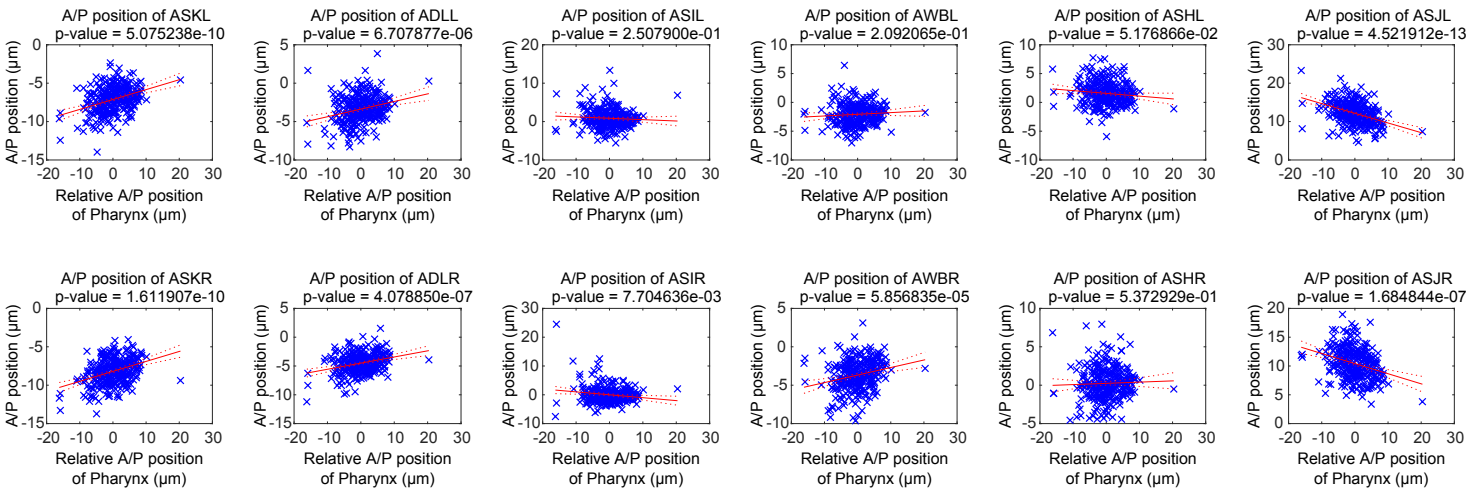

B: D/V position

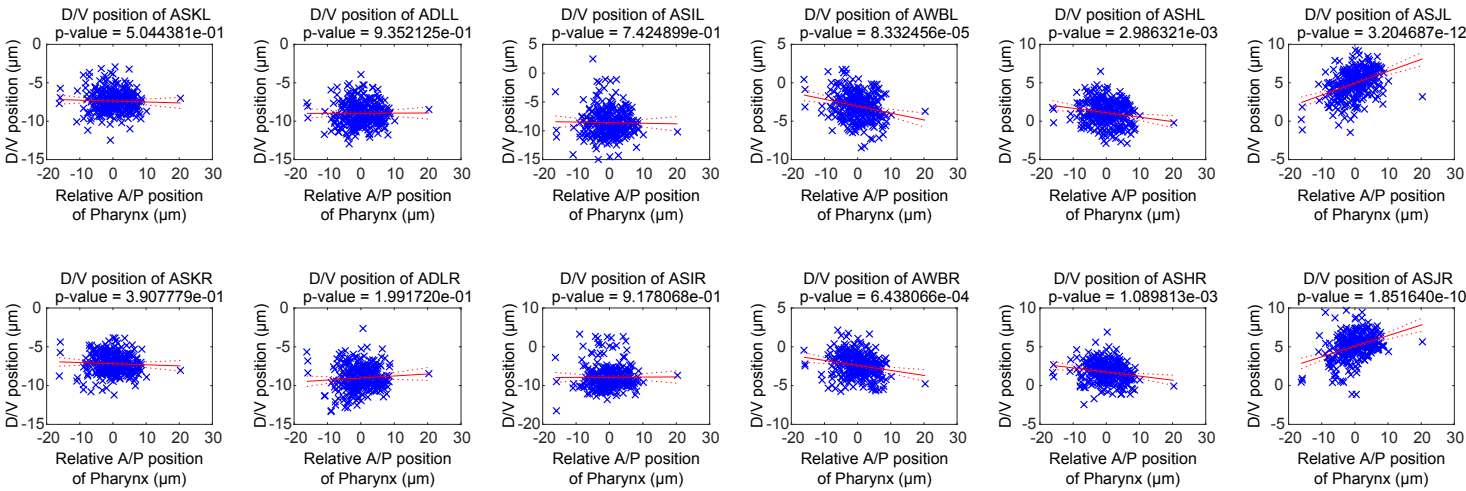

C: L/R position

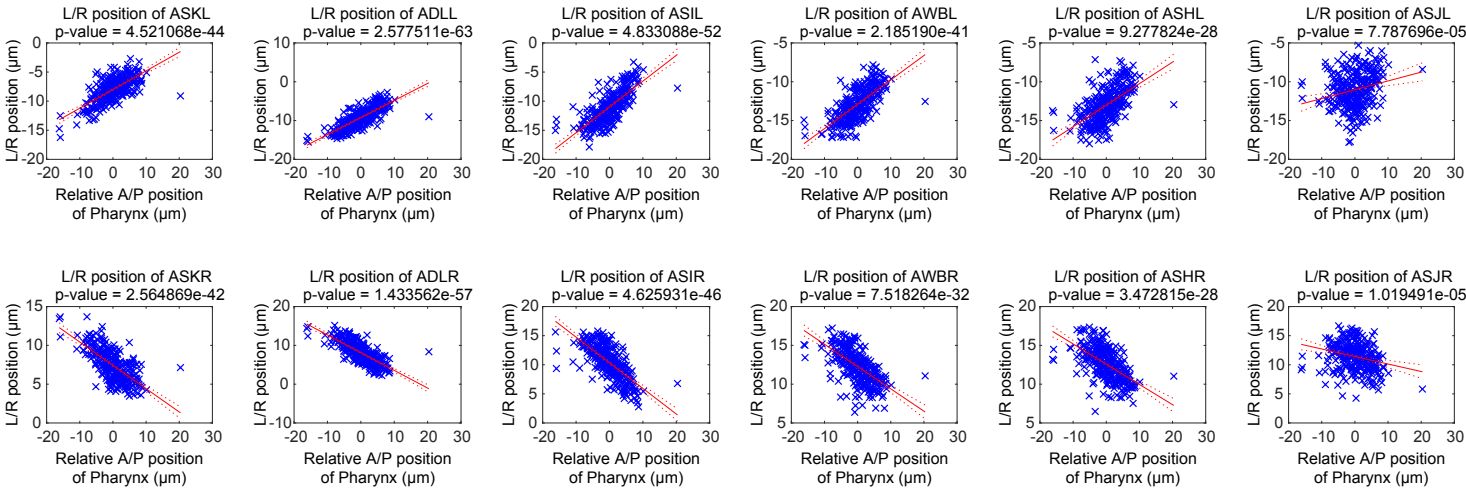

Supplement: Supplementary file 10 — Additional file 10: Figure S7. Position of posterior pharyngeal bulb affects cell positions. (A-C) A/P (A), D/V (B) and L/R (C) positions of dye positive cells plotted against relative A/P positions of pharynx. The relative A/P positions of pharynx were calculated from the mean difference of positions of pharyngeal cells from reference. Blue crosses indicate the cell positions in respective animals. The red lines and the red dotted lines indicate regression lines and 95% confidence bounds, respectively. The summary statistics are in Additional file 11: Table S4. [file 12915_2020_745_MOESM10_ESM.pdf]

A

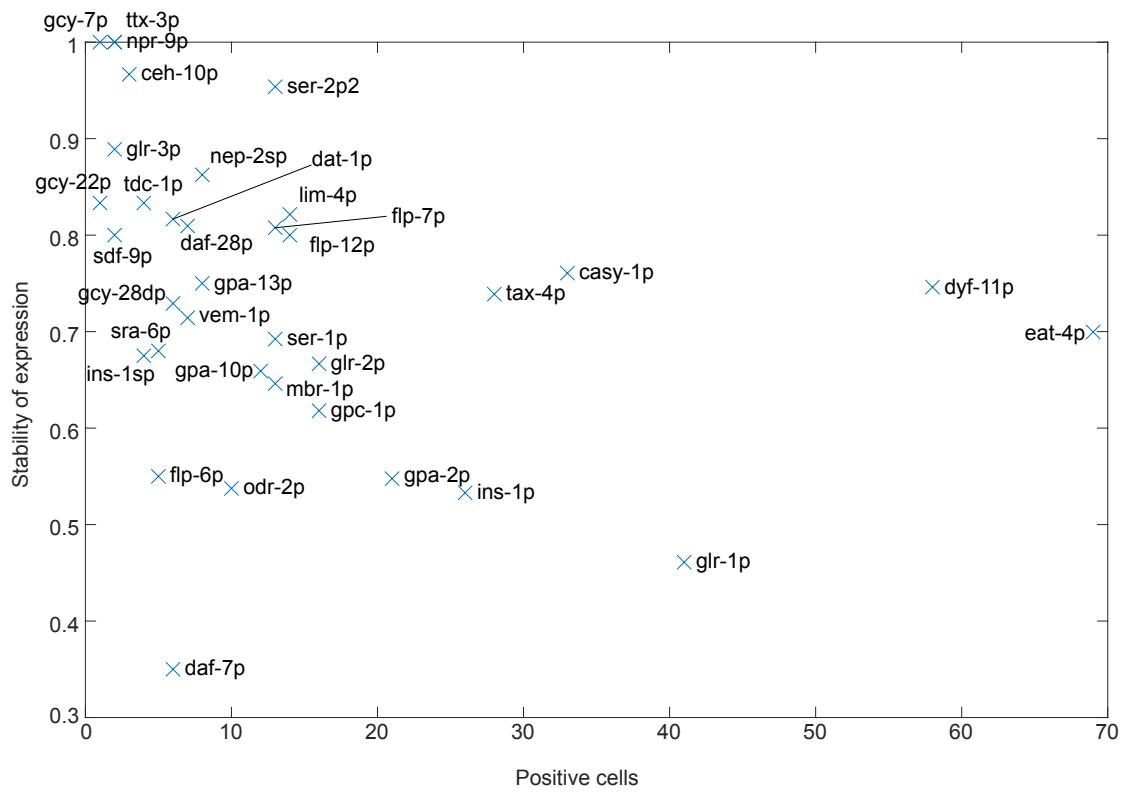

B

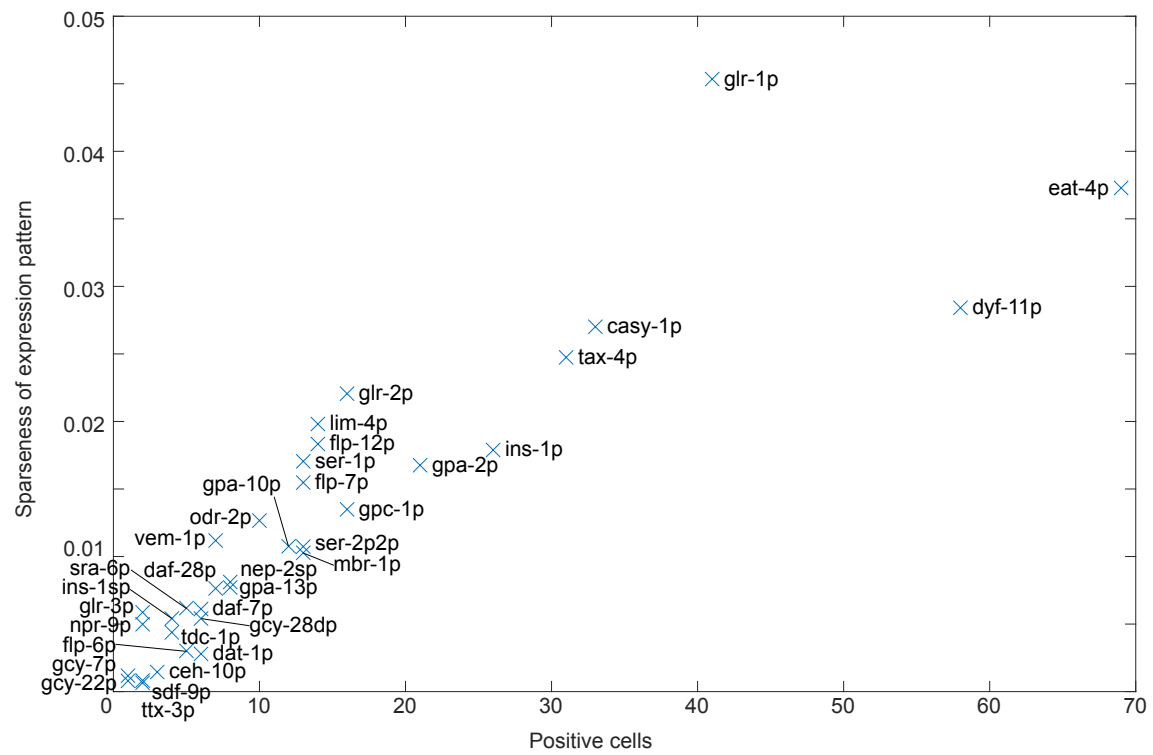

Supplement: Supplementary file 12 — Additional file 12: Figure S8. Stability and sparseness of expression pattern. (A) Number of positive cells and stability of expression of the cell-specific promoters. Same as Fig. 3a but fully labeled. (B) Number of positive cells and sparseness of expression pattern of the cell-specific promoters. Same as Fig. 3b but fully labeled. [file 12915_2020_745_MOESM12_ESM.pdf]

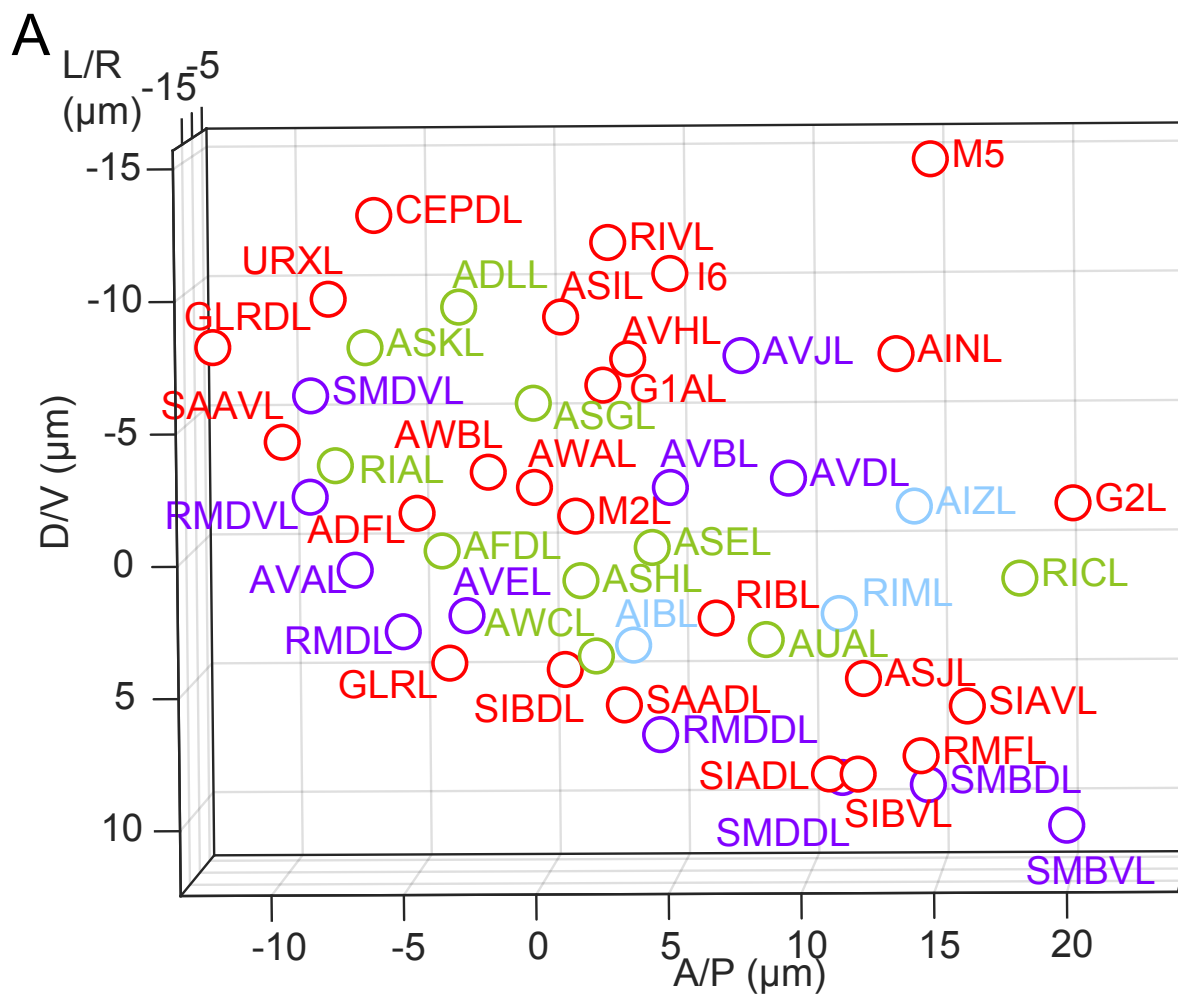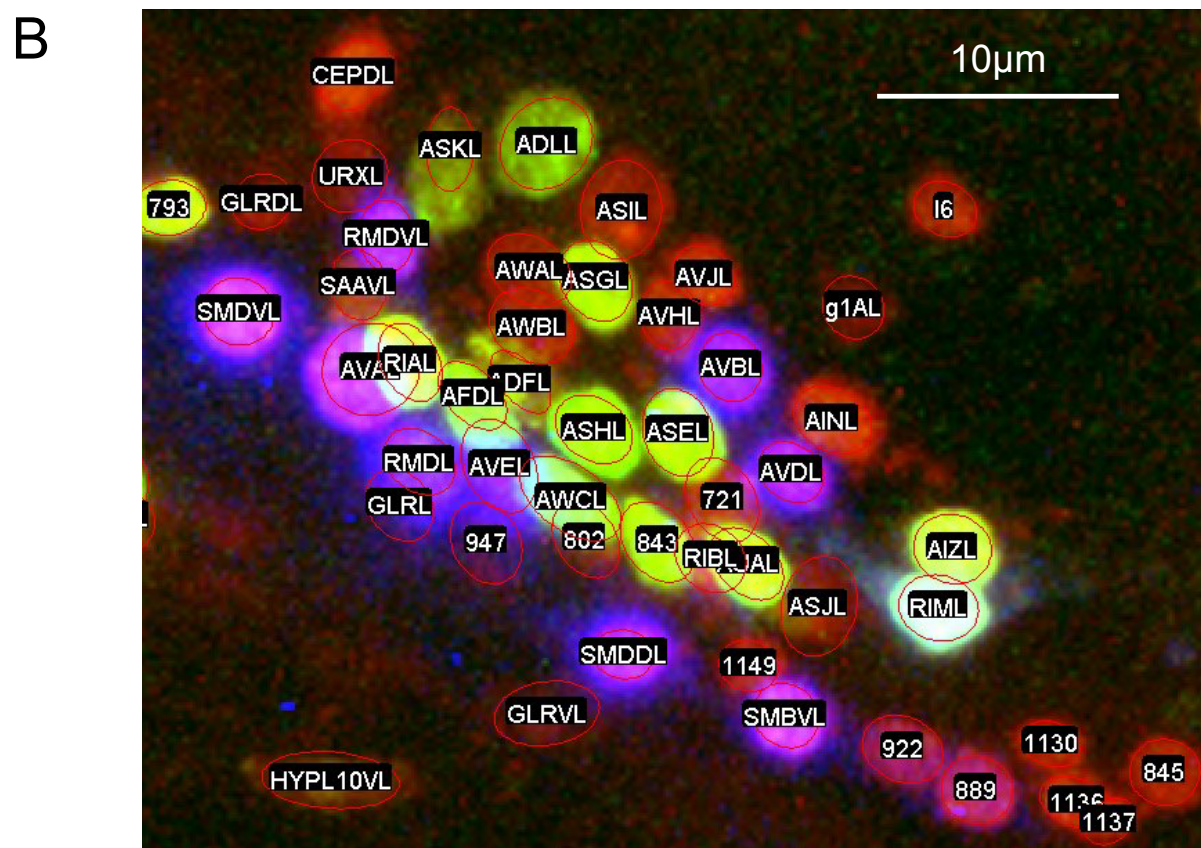

H20p (pan-neuronal) / eat-4p / glr-1p + ser-2p2

Supplement: Supplementary file 14 — Additional file 14: Figure S9. An example fluorescent image of JN3039 strain and annotated cell names. Zoomed version of Fig. 3d-e for visibility. [file 12915_2020_745_MOESM14_ESM.pdf]

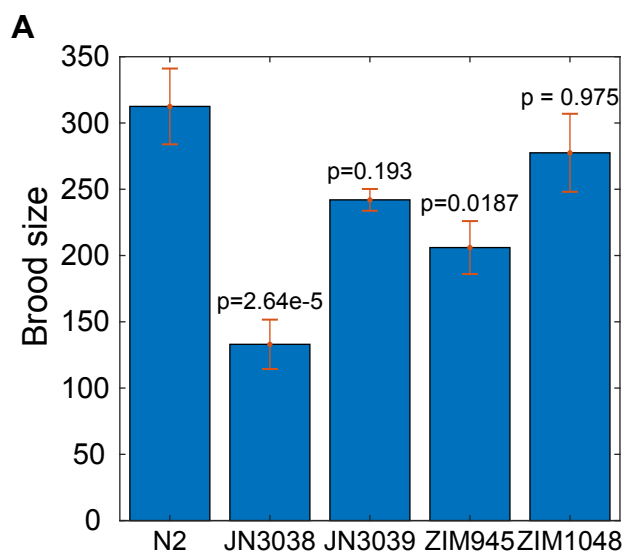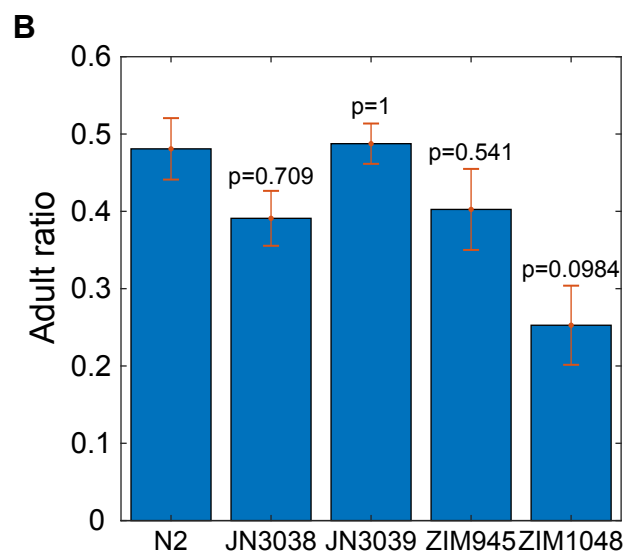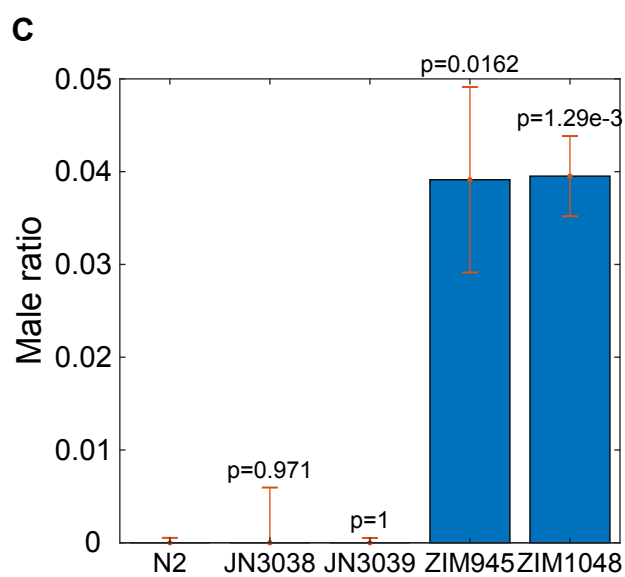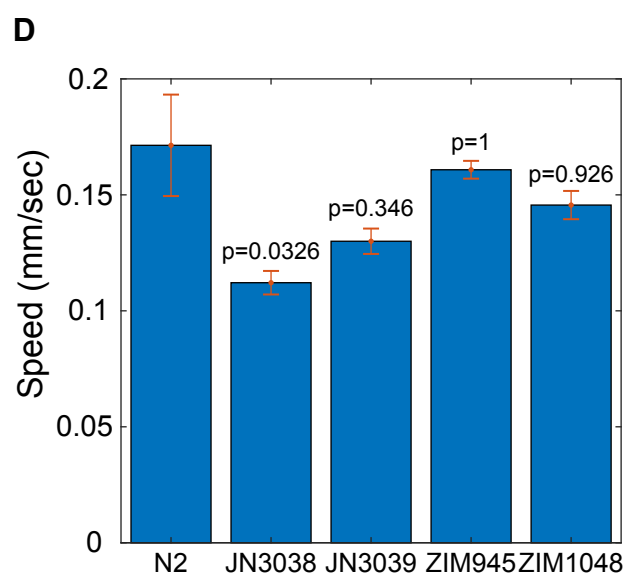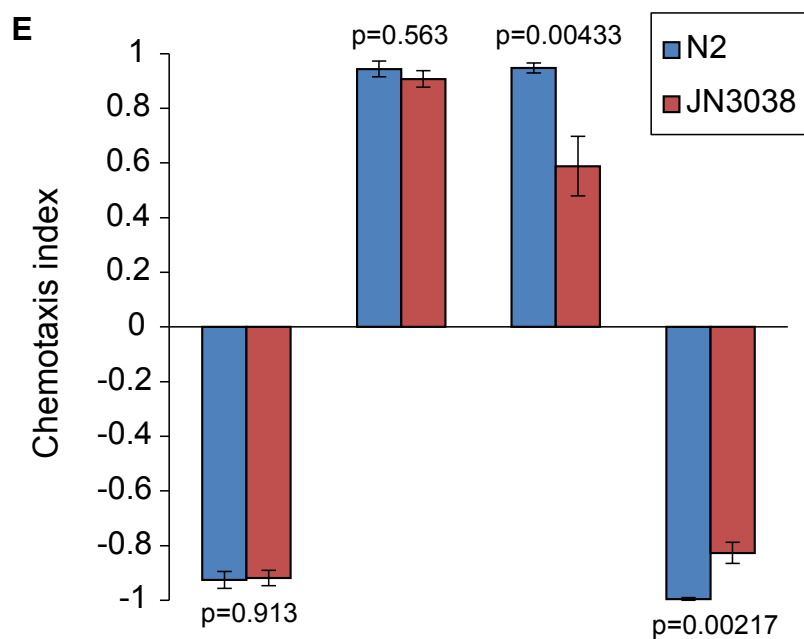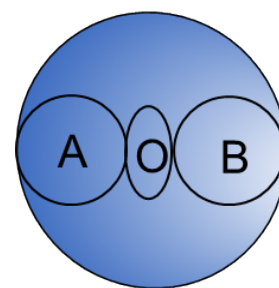

Chemotaxis index

$$= \frac{n_A - n_B}{n_{\text{Total}} - n_O}$$

|      |      |       |      |       |
|------|------|-------|------|-------|
| NaCl | 25mM | 100mM | 25mM | 100mM |
| Food | +    | +     | -    | -     |

Supplement: Supplementary file 16 — Additional file 16: Figure S10. Health of 4D strains. (A) Brood size of 4D strains including JN3038, JN3039, ZIM945 and ZIM 1048 were compared to N2. The animals were counted at 96 h after the parent animal was put on the plate (see Methods). The medians and the standard errors are shown. The sample size are n = 8 plates for N2, n = 9 plates for JN3038, n = 10 plates for JN3039, n = 11 plates for ZIM945, and n = 10 plates for ZIM 1048. The p-values of Tukey’s post hoc test following one-way ANOVA (Kruskal-Wallis test) to N2 are shown. The summary statistics are in Additional file 17: Table S6. (B) The ratio of adult hermaphrodite in (A). (C) The ratio of adult male in (A). (D) The locomotion speed of adult animals (see Methods). The means and the standard errors for n = 5 assays are shown. The p-values of Tukey’s post hoc test following one-way ANOVA (Kruskal-Wallis test) to N2 are shown. (E) Chemotaxis assay of JN3038. N2 and JN3038 animals were cultivated at 25 mM or 100 mM of NaCl with or without food. The conditioned animals were placed in the center of the assay plate with a NaCl gradient from 35 to 95 mM. The animals migrate to either side of the plate and chemotaxis index was calculated. A high chemotaxis index means the worms migrated to a high salt region. Mean ± SEM for n = 6 assays are shown. The p-values were obtained from a statistical test between JN3038 and N2 at the same condition (Wilcoxon rank sum test). [file 12915_2020_745_MOESM16_ESM.pdf]

Variation of relative position, atlas

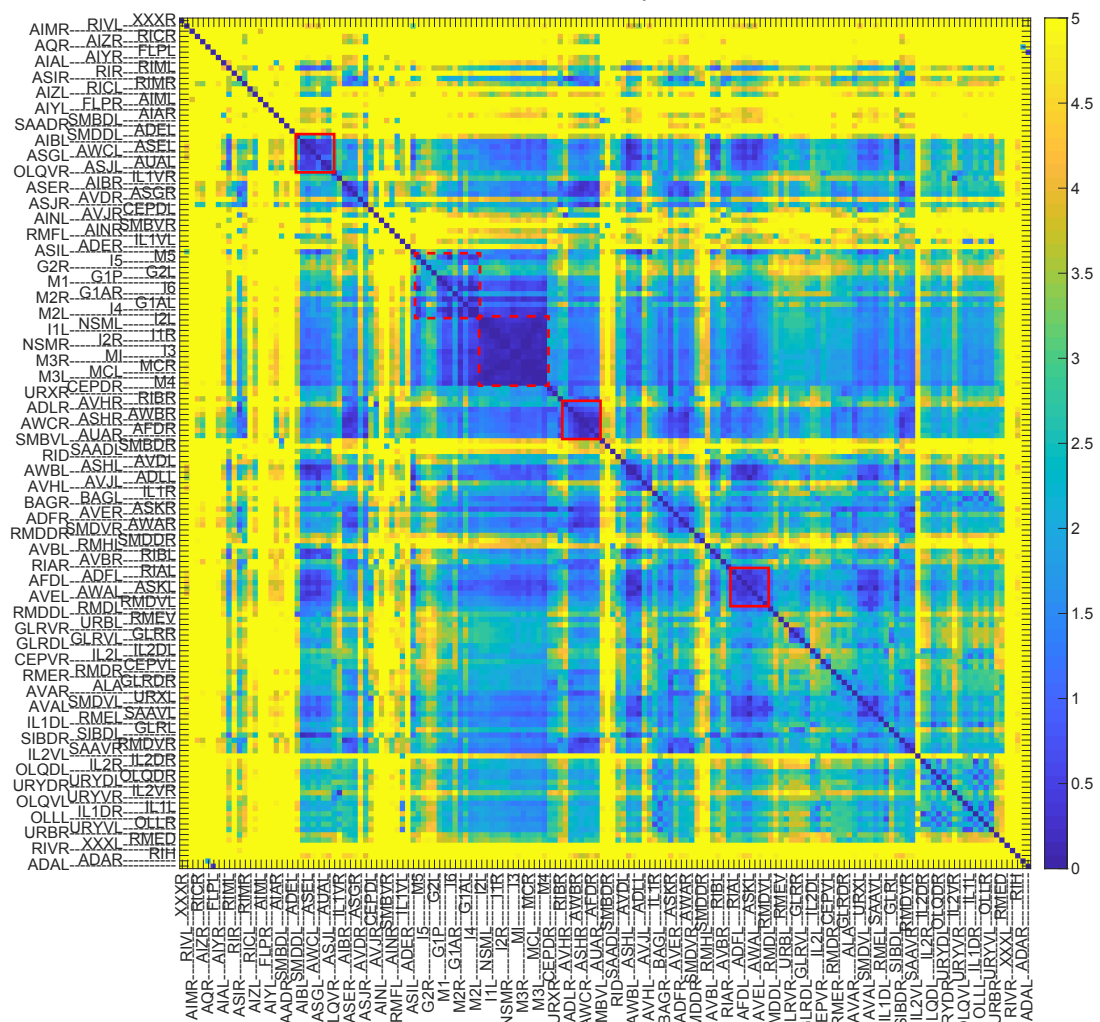

Supplement: Supplementary file 19 — Additional file 19: Figure S12. Variation of relative position of cell pairs. Variation of relative position of cell pairs. Orders of cells and colors are the same as in Fig. 2c. [file 12915_2020_745_MOESM19_ESM.pdf]

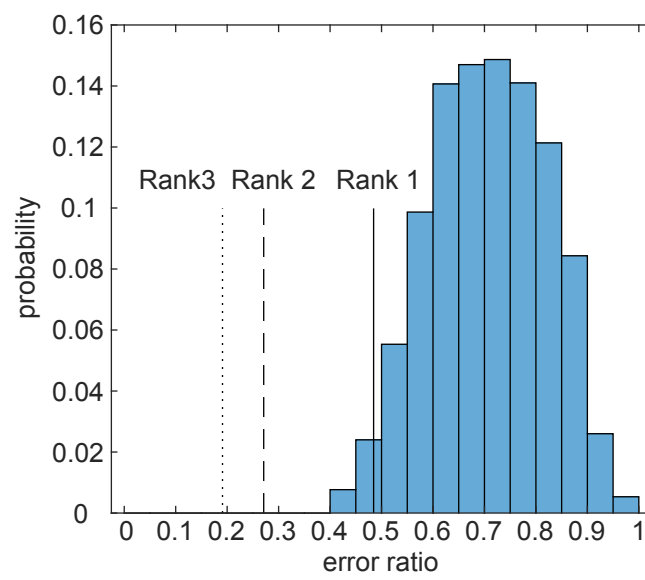

Supplement: Supplementary file 20 — Additional file 20: Figure S13. Error rate of each bipartite matching and majority voting. Error rate of each bipartite matching and majority voting are shown in the blue histogram and the black lines, respectively. The names of the cells were estimated based on their positions. The error rate was calculated as 1 – (Ncorrect)/(Nannotated) for each animal, where Nannotated is the number of human-annotated cells (ground truth) and Ncorrect is the number of cells whose annotation by the algorithm was correct. Cells un-annotated by human were not included in the calculation of error rate. The rank R indicates that it was considered correct if the correct annotation appeared in the top R estimations by the algorithm. [file 12915_2020_745_MOESM20_ESM.pdf]

Relationship between error rate of automatic annotation and detected count in the dataset

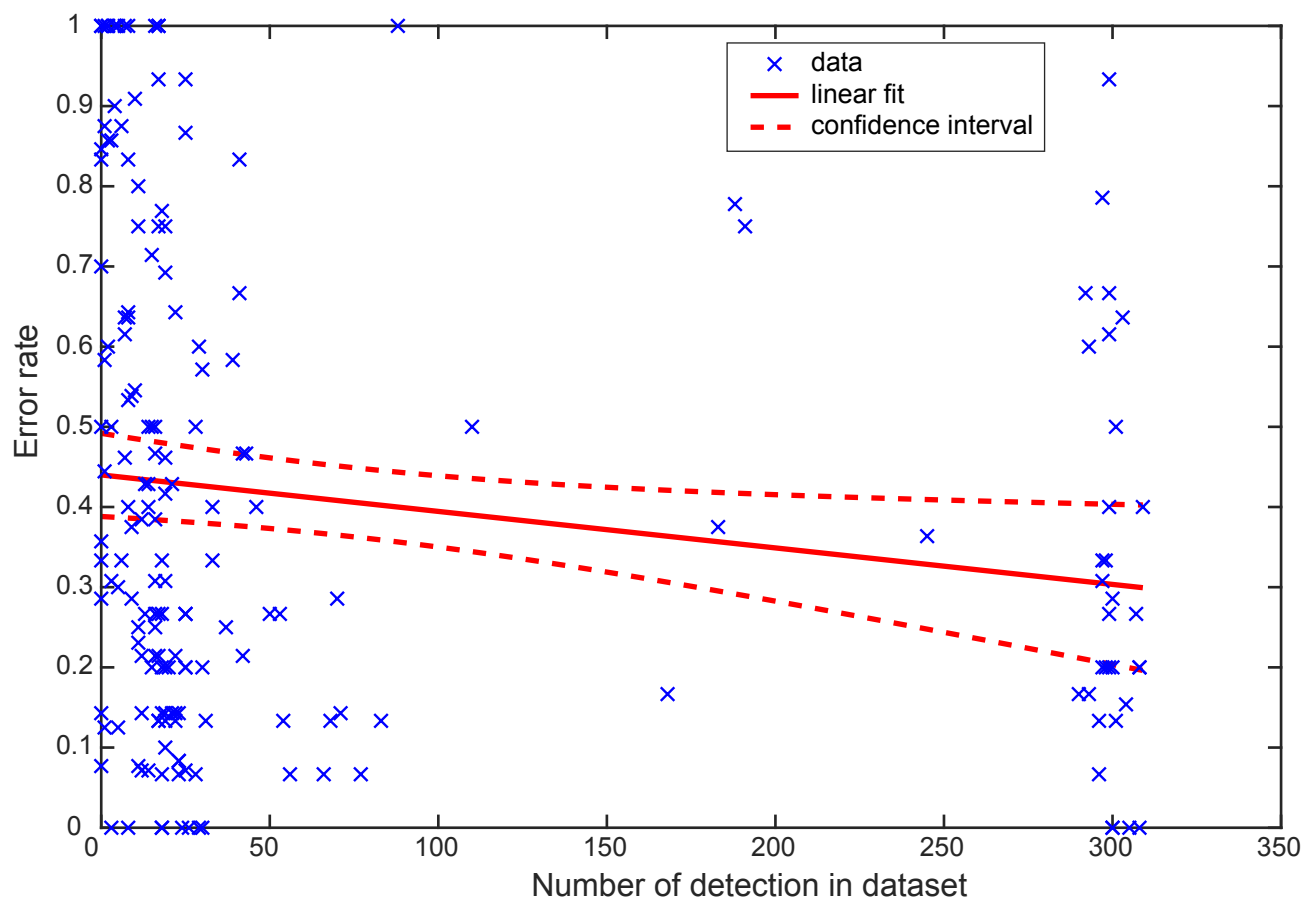

Supplement: Supplementary file 21 — Additional file 21: Figure S14. Relationship between error rate of automatic annotation for JN3039 and detected count in the neuron ID dataset. The error rate of automatic annotation for cells identified in JN3039 and detected count of the cell in the neuron ID are shown. The red lines and the red dotted lines indicate regression lines and 95% confidence bounds, respectively. The slope is − 4.55e-4 and the confidence interval is from − 0.0659 to 0.0169. The p-value is 0.0247. [file 12915_2020_745_MOESM21_ESM.pdf]

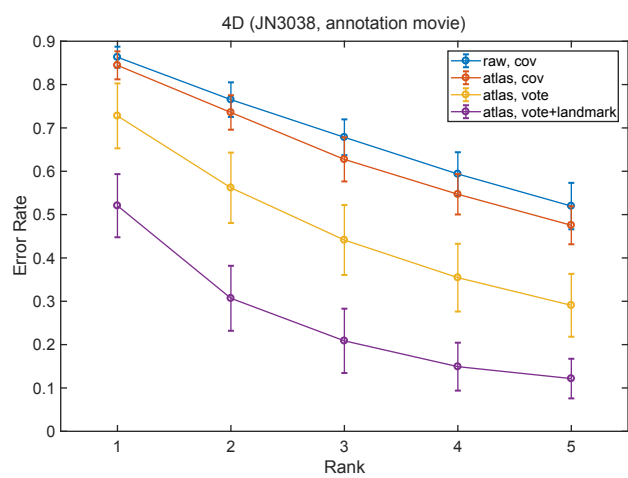

Supplement: Supplementary file 22 — Additional file 22: Figure S15. Error rates of the automatic annotation method for the animals in a microfluidic chip. Error rates of the automatic annotation method for the animals in a microfluidic chip for whole-brain activity imaging (JN3038 strain). Mean ± standard deviation over 12 animals are shown. [file 12915_2020_745_MOESM22_ESM.pdf]

A

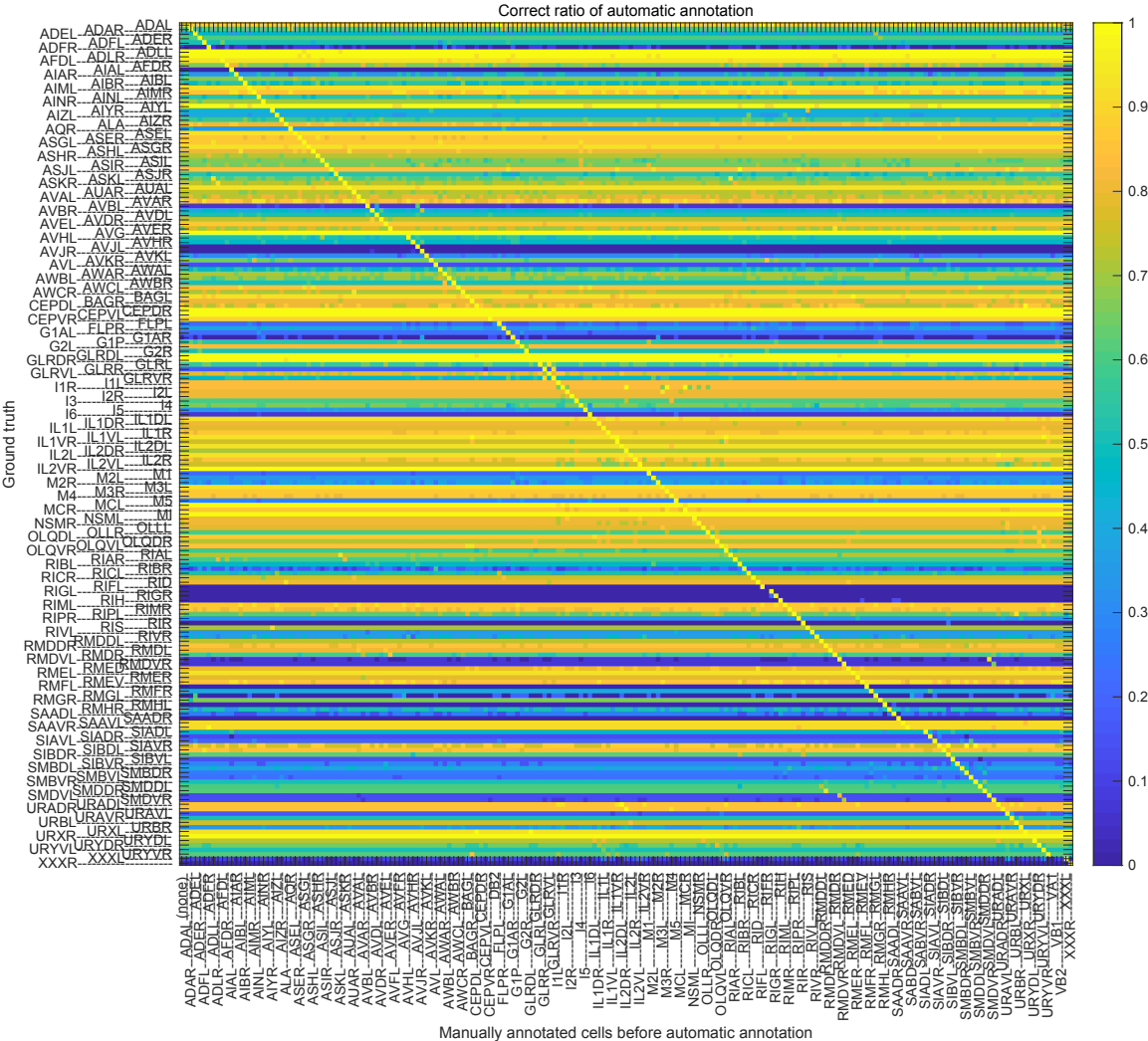

B

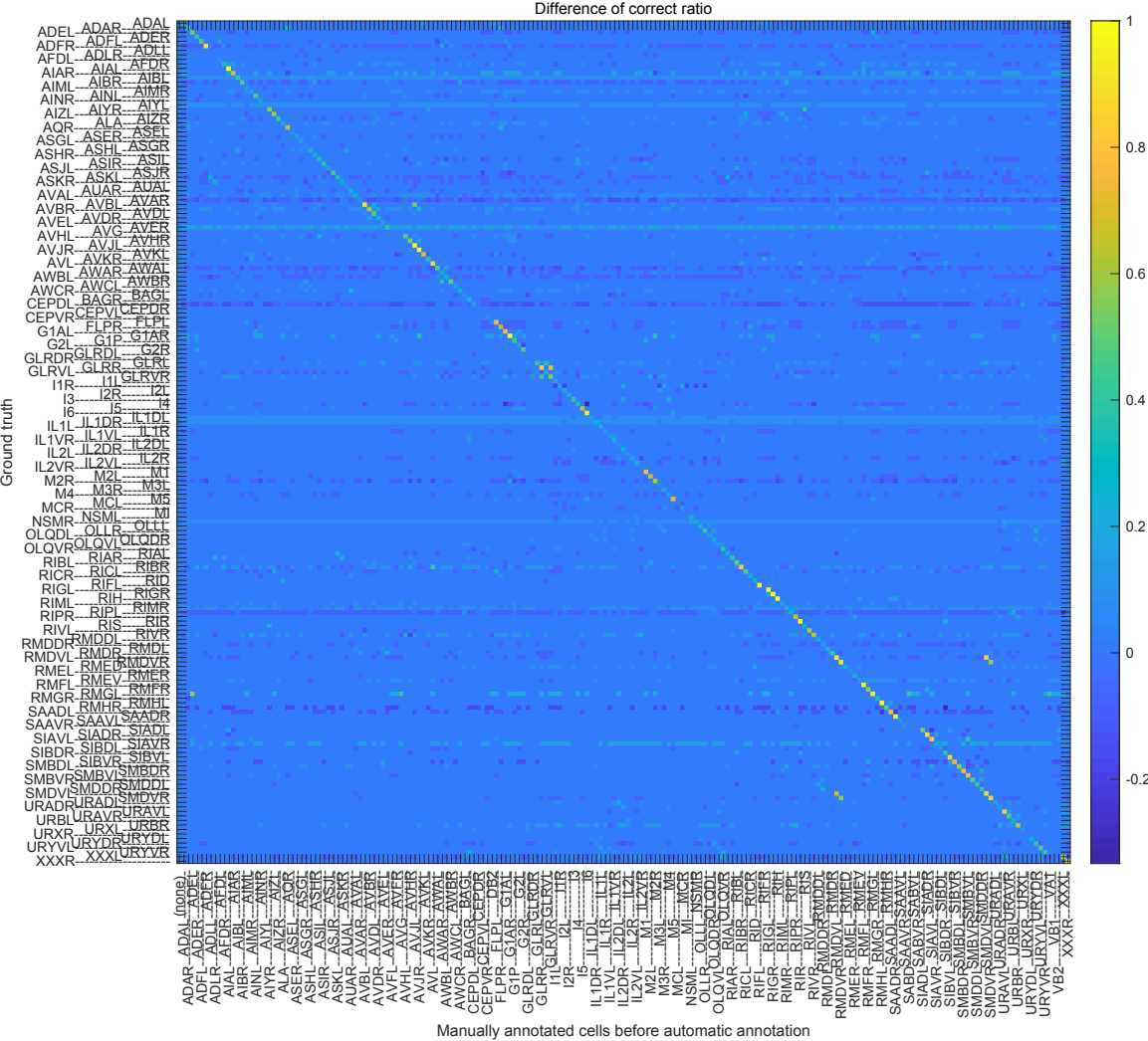

Supplement: Supplementary file 24 — Additional file 24: Figure S16. Correct rate of automatic annotation and its improvement by manual annotation. (A) The effect of prior manual annotation on the correct rate of automatic annotation. The annotation of a single cell type was specified manually before performing the automatic annotation. The error rates of automatic annotation for cells identified in JN3039 are shown. (B) Improvement of correct rate was obtained by subtracting the original correction rate from the correction rate with prior manual annotation. [file 12915_2020_745_MOESM24_ESM.pdf]
