# Supplementary material for: Neuron ID dataset facilitates neuronal annotation for whole-brain activity imaging of C. elegans
Source: BMC Biol. 2020 Mar 19;18:30. doi: 10.1186/s12915-020-0745-2 (PMC7081613; doi:10.1186/s12915-020-0745-2)
Supplement: Supplementary file 27 — Additional file 27: Note S1. Optimization of parameters for atlas generation. [file 12915_2020_745_MOESM27_ESM.docx]

Supplementary Note 1: Optimization for parameters in atlas generation

The generated reference samples serve as a set of virtual atlases that imitate observed topological variations of cellular positions across different worm samples. To obtain more realistic atlases, we optimized $\Sigma=\text{diag}\left( \sigma_{1},\sigma_{2},\sigma_{3} \right)$ in Eq 1, which is the parameter to control the smoothness of displacements in the sequential alignments. First, we designed a measure $\boldsymbol{J}_{\boldsymbol{t\to s}}\boldsymbol{(i)}$ to characterize the coherency of the displacement of cell $i$ with respect to its neighboring cells in two images, $I_{t}$ and $I_{s}$:

$\boldsymbol{J}_{\boldsymbol{t\to s}}\left( \boldsymbol{i} \right)\boldsymbol{=}{\min_{\boldsymbol{r}} \left\langle\boldsymbol{d}_{\boldsymbol{i}}\boldsymbol{-r,}{\bar{\boldsymbol{d}}}_{\mathcal{N}_{\boldsymbol{i}}}\boldsymbol{-r} \right\rangle\boldsymbol{\equiv}\left\| \boldsymbol{d}_{\boldsymbol{i}}\boldsymbol{-}{\bar{\boldsymbol{d}}}_{\boldsymbol{M}_{\boldsymbol{i}}} \right\|}^{\boldsymbol{2}}$, where $\left\langle\boldsymbol{\cdot} \right\rangle\boldsymbol{=}\mathbf{dot product}$. (2)

The displacement of cell $i$ was $\boldsymbol{d}_{i}=\boldsymbol{x}_{i}^{s}-\boldsymbol{x}_{i}^{t}$. The mean displacement of its neighbor set $M_{i}$ was calculated as ${\bar{\boldsymbol{d}}}_{M_{i}}=\left| M_{i} \right|^{-1}\sum_{j\in M_{i}} \boldsymbol{d}_{j}$ where $M_{i}$ denotes a set of cells neighboring within the squared distance less than 70 px with respect to cell $i$ in the image $I_{t}$. For each cell, this measure was calculated for all pairs of the 311 human annotation data if the number of neighboring cells was larger than four. In addition, we calculated $\boldsymbol{J}_{\boldsymbol{t\to s}}\left( \boldsymbol{i} \right)$ for randomly chosen 1,000 pairs of the computationally manipulated atlases. Finally, we used Bayesian optimization technique to minimize the Kullback-Liebler divergence between the normal distributions fitted to given $\boldsymbol{J}_{\boldsymbol{t\to s}}\left( \boldsymbol{i} \right)$ for the human annotation data and the computationally manipulated atlases, respectively.
