# Supplementary material for: Neuron ID dataset facilitates neuronal annotation for whole-brain activity imaging of C. elegans
Source: BMC Biol. 2020 Mar 19;18:30. doi: 10.1186/s12915-020-0745-2 (PMC7081613; doi:10.1186/s12915-020-0745-2)
Supplement: Supplementary file 9 — Additional file 9: Figure S6. Less varying neuron pairs. (A) Less varying neuron pairs were obtained by random permutation of animals (see Methods) and the less varying pairs in the left half of the body are shown by red lines. The pairs including pharyngeal cells were omitted for visualization. (B) The less varying pairs including pharyngeal cells are shown by red lines. [file 12915_2020_745_MOESM9_ESM.pdf]

A

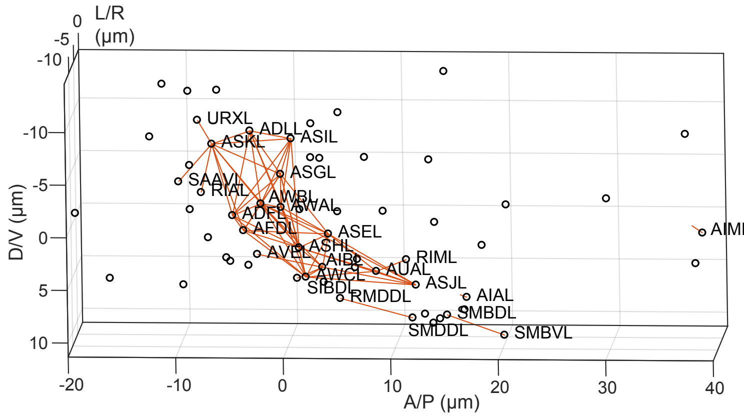

L/R (μm)
D/V (μm)
A/P (μm)
URXL
ADLL
ASIL
ASKL
ASGL
SAAVL
RIAL
AWBL
AWAL
ADFL
AFDL
ASEL
ASHL
AVBL
AIBL
AWCL
AUAL
RIML
ASJL
SIBDL
RMDDL
AIAL
SMBDL
SMDDL
SMBVL
AIML

B

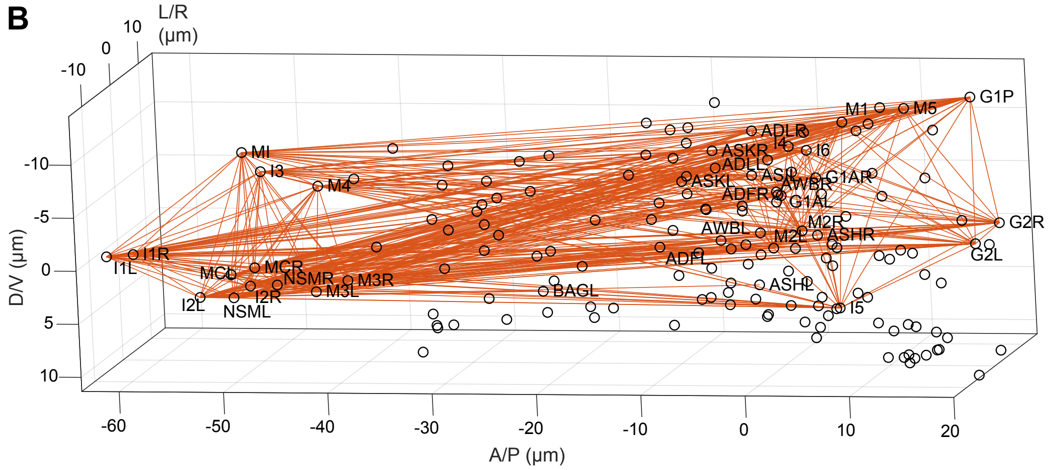

L/R (μm)
D/V (μm)
A/P (μm)
MI
I3
M4
I1L
I1R
MCL
MCR
NSMR
M3R
M3L
I2L
I2R
NSML
BAGL
M1
M5
G1P
ADLR
ASKR
I4
I6
ADLL
ASKL
ASIL
AWBR
G1AR
ADFR
G1AL
M2R
G2R
AWBL
M2L
ASHR
G2L
ADFL
ASHL
I5
