# Supplementary material for: Neuron ID dataset facilitates neuronal annotation for whole-brain activity imaging of C. elegans
Source: BMC Biol. 2020 Mar 19;18:30. doi: 10.1186/s12915-020-0745-2 (PMC7081613; doi:10.1186/s12915-020-0745-2)
Supplement: Supplementary file 18 — Additional file 18: Figure S11. Comparison of the synthetic atlas and the neuron ID dataset. (A) Visualization of the variation of the cell positions in synthetic atlas. The cells and colors are the same as Fig. 2a. (B) Distance of mean position of cells between the atlas and the neuron ID dataset. (C) Ratio of volume of ellipsoid (covariance of the positions of the cell) between the atlas and the neuron ID dataset. (D) Comparing the variation of relative positions of dataset and that of atlas. The color indicates how many times the neuron pair is co-detected in an animal of the neuron ID dataset. [file 12915_2020_745_MOESM18_ESM.pdf]

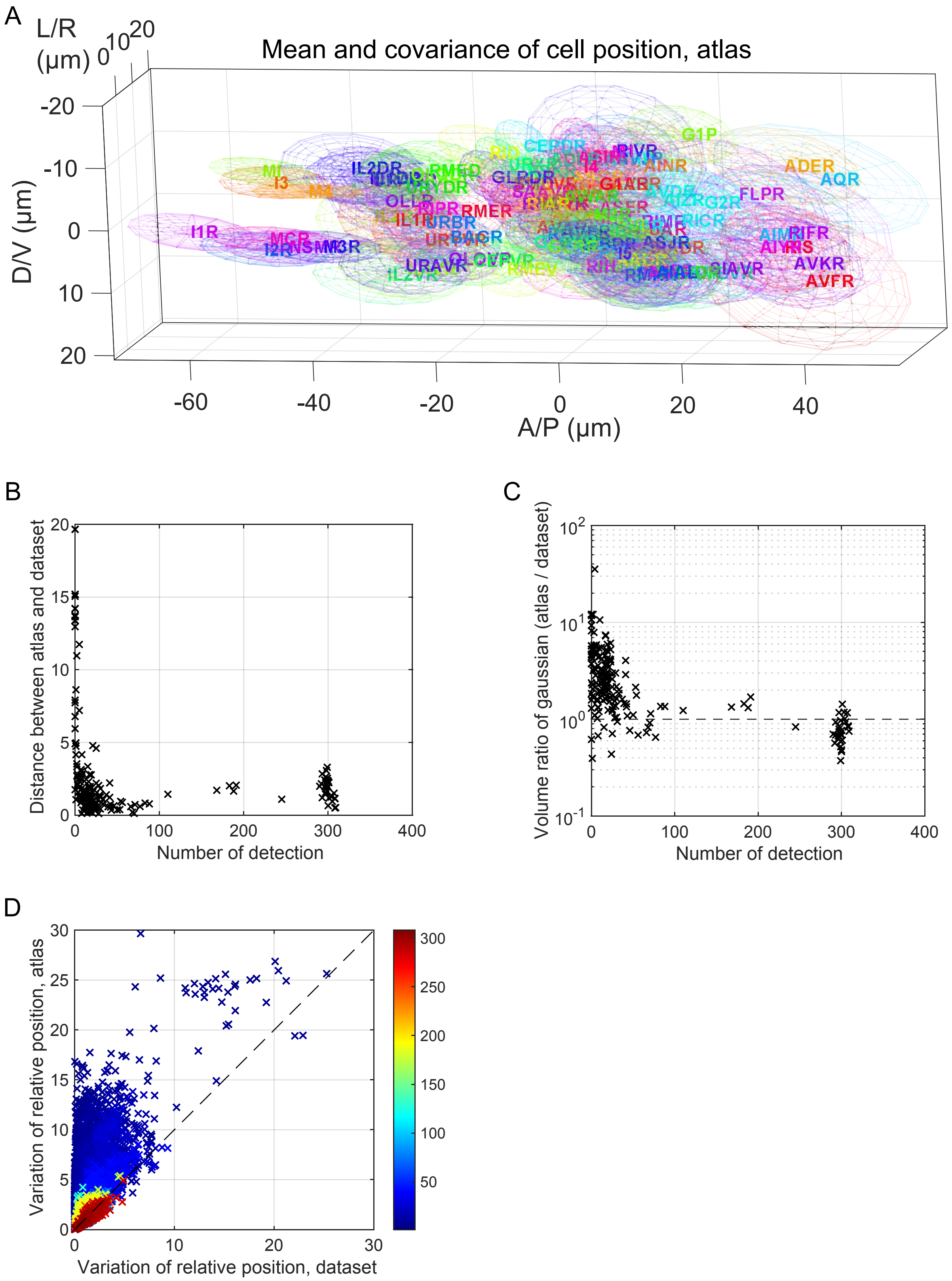
A
Mean and covariance of cell position, atlas
L/R (μm)
D/V (μm)
A/P (μm)
B
Distance between atlas and dataset
Number of detection
C
Volume ratio of gaussian (atlas / dataset)
Number of detection
D
Variation of relative position, atlas
Variation of relative position, dataset
