# Supplementary material for: Neuron ID dataset facilitates neuronal annotation for whole-brain activity imaging of C. elegans
Source: BMC Biol. 2020 Mar 19;18:30. doi: 10.1186/s12915-020-0745-2 (PMC7081613; doi:10.1186/s12915-020-0745-2)
Supplement: Supplementary file 25 — Additional file 25: Figure S17. Most valuable cells for improving accuracy of automatic annotation. (A) Improvement of correct rate for prior annotated cell itself. Difference of correct rate is 0.9 when the correct rate of the cell in original case (i.e. without prior annotation) is 0.1. (B) Mean improvement of correct rate for the cells other than the prior annotated cells. Prior annotation of SMDVL achieved about 0.59% improvement of correct rate, indicating that 1.1 cells were corrected in addition to SMDVL. (C) Mean improvement of correct rate through all cells. Prior annotation of SMDVL achieved about 1.1% improvement of correct rate, indicating that 2.0 cells were corrected. [file 12915_2020_745_MOESM25_ESM.pdf]

A

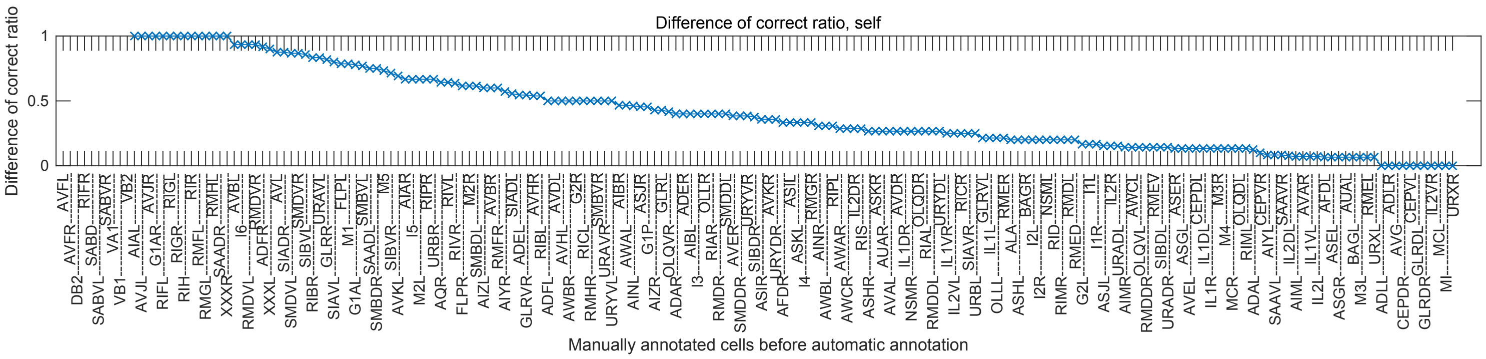
Difference of correct ratio, self
Difference of correct ratio
Manually annotated cells before automatic annotation

B

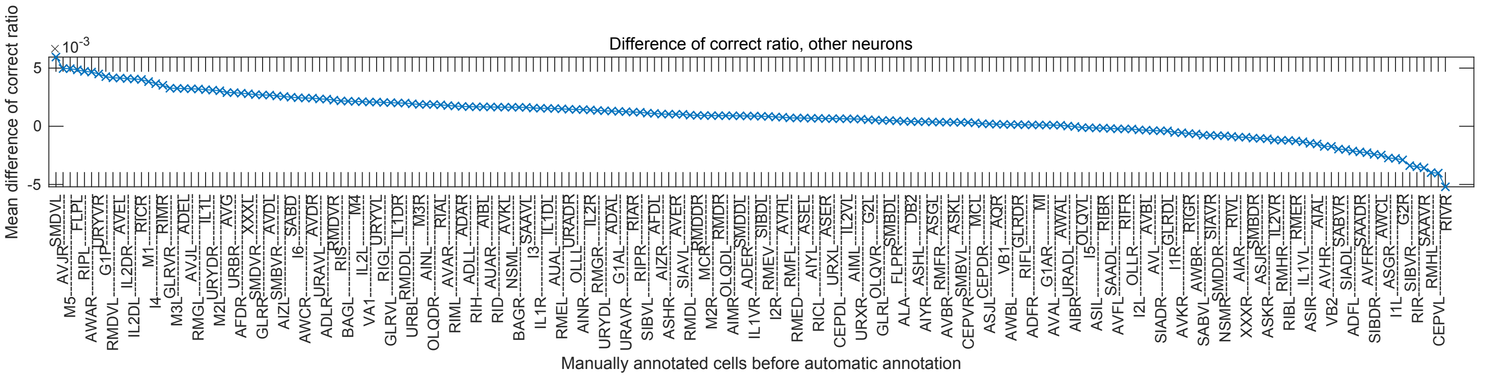
Difference of correct ratio, other neurons
Mean difference of correct ratio
Manually annotated cells before automatic annotation

C

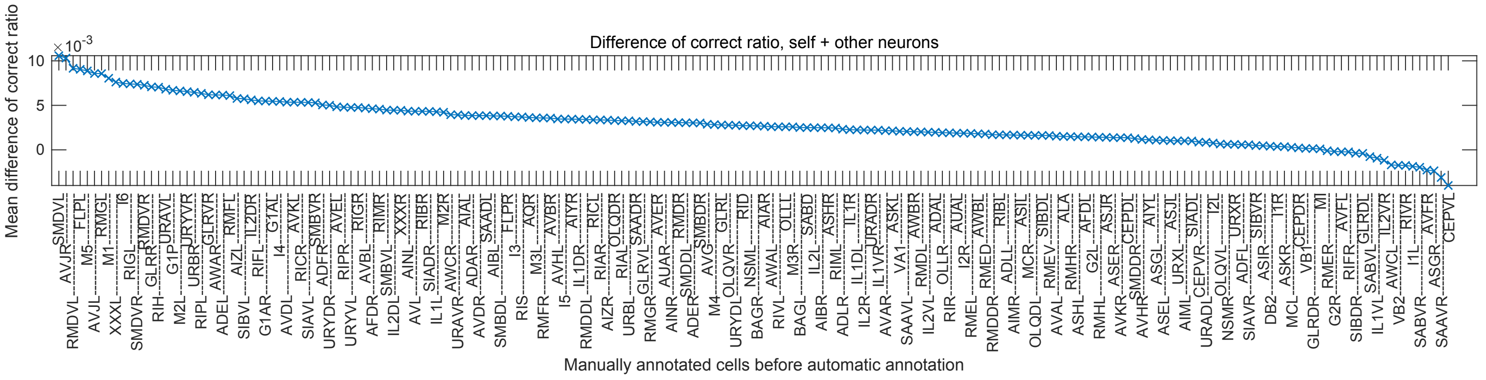
Difference of correct ratio, self + other neurons
Mean difference of correct ratio
Manually annotated cells before automatic annotation
